# Preparation and Applications of Organo-Silica Hybrid Mesoporous Silica Nanoparticles for the Co-Delivery of Drugs and Nucleic Acids

**DOI:** 10.3390/nano10122466

**Published:** 2020-12-09

**Authors:** Iris Pontón, Andrea Martí del Rio, Marta Gómez Gómez, David Sánchez-García

**Affiliations:** Grup d’Enginyeria de Materials (GEMAT), Institut Químic de Sarrià (IQS), Universitat Ramon Llull (URL), Via Augusta, 390, 08017 Barcelona, Spain; irispontonb@iqs.edu (I.P.); andreamartid@iqs.url.edu (A.M.d.R.); martagomezg@iqs.url.edu (M.G.G.)

**Keywords:** mesoporous silica, nanoparticles, drug delivery, co-delivery, combination therapies, RNA

## Abstract

Combination therapies rely on the administration of more than one drug, with independent mechanisms of action, aiming to enhance the efficiency of the treatment. For an optimal performance, the implementation of such therapies requires the delivery of the correct combination of drugs to a specific cellular target. In this context, the use of nanoparticles (NP) as platforms for the co-delivery of multiple drugs is considered a highly promising strategy. In particular, mesoporous silica nanoparticles (MSN) have emerged as versatile building blocks to devise complex drug delivery systems (DDS). This review describes the design, synthesis, and application of MSNs to the delivery of multiple drugs including nucleic acids for combination therapies.

## 1. Introduction

The fight against complex diseases requires novel strategies and innovative approaches. The emergence of nanotechnologies has offered a wide range of new solutions with the promise to improve current treatments. The selective delivery of drugs is one of the aspects that limits the effectiveness of the treatment of many diseases. Furthermore, in some cases the administration of multiple drugs, is necessary, to overcome resistance, such is the case of some cancers or tuberculosis [1,2,3,4,5]. Therefore, there is an urgent need of drug delivery systems (DDS) which allow the selective delivery of multiple drugs [6]. In this context, mesoporous silica nanoparticles (MSN) have emerged as versatile building blocks to devise complex DDSs [7,8,9,10,11,12].

The objective of this review is to offer an overview of the applications of functionalized MSNs in the delivery of multiple drugs and nucleic acids. Excellent reviews on the topic have been published with special emphasis in the medical application [13,14,15,16]. The present work aims to describe and examine the different DDSs from a synthetic point of view. The literature reviewed covers from 2009 to 2020.

This review is broken into three parts. First, a brief introduction on the preparation and application of MSNs in the medical field. The following part is devoted to DDSs based on MSNs designed for multiple drug delivery. This part classifies the DDSs in the following types: MSNs with drugs grafted inside the pores, MSNs containing a drug inside the pores and a second drug outside as stopper, DDSs containing a drug inside the pores using cyclodextrins (CD) as valves, and DDSs loaded with one drug and coated with a polymer. Finally, in the last section the application of MSNs for the delivery of combination of drugs and nucleic acids, especially RNA, will be discussed. In this part, the nano-vehicles are divided into three types: DDSs with siRNA attached covalently onto the surface of the MSNs, MSNs coated by a polycationic polymer, and MSNs containing RNA inside the pores.

## 2. Mesoporous Silicas in Drug Delivery

**Figure 1:**
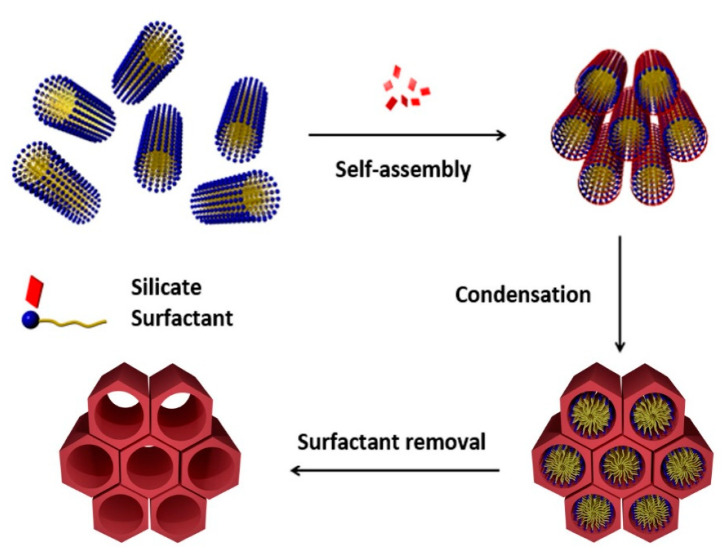
Preparation of MCM-41 following a modification of the Stöber method. Reproduced from reference [24] https://doi.org/10.3390/catal8120617 with permission of MDPI, 2018.

The preparation of MSNs was independently reported in 1990 by researchers of Waseda University (Japan) and in 1992 by the Mobil Oil Corporation [8,17,18]. Among all mesoporous materials prepared to date, such as HMM-33 [19] and SBA-15 [20]; MCM-41 (MCN, Mobil Composition of Matter) have become one of the most widely used for biological applications [21]. This type of nanoparticles is usually synthesized following a modification of the Stöber method, which consists in the condensation of silica precursors, typically sodium silicate or tetraethyl orthosilicate (TEOS), in the presence of cationic surfactants such as cetyltrimethylammonium bromide (CTAB), that act as structure-directing template in basic conditions (pH = 11) and temperatures in the range of 30–60 °C (Figure 1) [17,22,23].

MSNs are characterized by a high surface area (>900 m^2^/g), large pore volume (>0.9 cm^3^/g), and tunable pore size with a narrow distribution (2–10 nm) [25]. Furthermore, these materials show excellent chemical and thermal stability. However, it is its intrinsic low toxicity and biocompatibility that make MSNs versatile and promising platforms for drug delivery.

The first report on the use of MSNs as DDSs was presented by the Vallet–Regí group in 2001 [26,27]. In this pioneering work, MCM-41 were loaded with ibuprofen and its release was studied in a simulated body fluid. Since this seminal report, extensive work has been carried out specially to gain control upon the release of the cargo [18]. To this end, stimuli-responsive MSNs have been engineered to release the drug upon precise conditions such as low pH, temperature, presence of enzymes, or high glutathione (GSH) concentration [28,29,30,31]. Thanks to this body of work, the chemistry of MSNs has matured enough to allow designing complex DDSs that selectively deliver combinations of multiple drugs including sensitive RNA strands.

The success of MSNs in drug delivery has prompted researchers to exploit other porous materials such as the closely related hollow silica nanospheres (HMSN) [32,33]. This nanomaterial possesses a large central hole and an external mesoporous silica shell. The central void is formed by removal of a spherical template similarly to the formation of the mesoporous phase in MSNs. This morphology offers advantages compared to MSNs such as higher loading capacity, lower density, and larger specific area [34].

## 3. Multiple Drug Delivery Systems Based on MSNs

Two general strategies have been envisioned to prepare MSNs charged with two different drugs: MSNs containing drugs attached covalently inside the pores and MSNs grafted with a drug inside the pores and the second one, being attached to the outer surface of the particle.

### 3.1. DDSs with Drugs Grafted inside the MSNs

The pores of the MSNs are ideal reservoirs for drugs. However, because the pores are open the release of the load must be controlled by means of a smart device sensitive to a stimulus. A simple way to minimize the leakage is the attachment of the drugs through a cleavable bond onto the inner surface of the particle.

**Figure 2:**
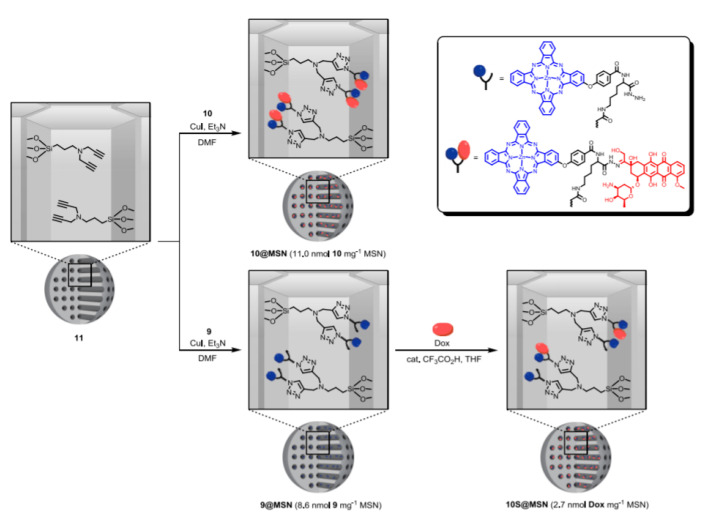
Two synthetic pathways for the preparation of mesoporous silica nanoparticles (MSN) containing conjugate doxorubicin (Dox)-zinc(II) phthalocyanine (ZnPc) inside the pores of the nanoparticles (NP). Reproduced from reference [35] https://doi.org/10.1002/chem.201703188. Copyright 2017 Wiley-VCH verlag GmbH & Co. KGaA, Weinheim.

An example was given by Ng and co-workers in 2017. This group prepared a DDS for the dual delivery of doxorubicin (Dox) and zinc(II) phthalocyanine (ZnPc) [35]. The system allows for exploring the synergism between Dox, a topoisomerase II inhibitor, and ZnPc a well-known photosensitizer for photodynamic therapy (PDT). In these nanoparticles, the drugs were introduced into the nano-channels forming part of a conjugate Dox-ZnPc tethered by an acid cleavable hydrazone linker containing an azido group. The conjugate was designed in such a way that only Dox was released from the particle under acidic conditions since the phthalocyanine was bonded to the linker by an amide group. Once in hand of the conjugate, the particles were synthetized in a two-steps process. First, the nanochannels were functionalized with a dialkyne chain by grafting. Then, the conjugate was anchored to the particles via a copper(I)-catalyzed azide-alkyne cycloaddition reaction. Alternatively, Dox can be introduced by exposure of the functionalized particles with ZnPc and a hydrazide moiety to a solution of the drug (Figure 2). Interestingly, the release of Dox under acidic conditions was studied by monitoring the reduction in the Förster resonance energy transfer (FRET) from the excited Dox to the ZnPc after acidic cleavage of the hydrazone linker, and the increase in fluorescence of the Dox released. In vitro studies carried out with hepatocellular carcinoma HepG2 cells demonstrated by fluorescence the successful delivery of the two drugs. According to the authors, Dox’s fluorescence suggests that the acidic intracellular environment triggered the release of the drug by cleaving the hydrazone linker.

**Figure 3:**
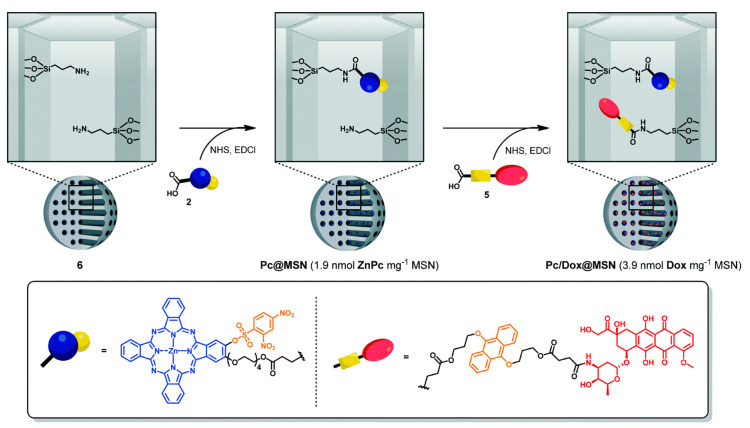
Preparation of Pc@MSN and Pc/Dox@MSN. Reproduced from reference [36] https://doi.org/10.1039/D0TB00636J with permission from The Royal Society of Chemistry.

The DDS described before bases its release mechanism on the acidity of the tumoral environment. Since the DDS was designed for PDT treatments, the same group developed a new variant of this nano-vehicle which is sensitive to singlet oxygen. This new DDS incorporates a singlet-oxygen-cleavable linker (9,10-dialkoxyanthracene) able to release Dox upon irradiation with red light [36]. In turn, the photosensitizer (ZnPc) was tethered to a 2,4-dinitrobenzenesulfonate moiety, which is GSH-sensitive and deactivates ZnPc (Figure 3). Thus, in the presence of GSH and upon irradiation with red light, the phthalocyanine units were activated by detaching from the quenching component to emit fluorescence and generate singlet oxygen. Although the system showed an excellent cytotoxicity upon irradiation, the authors noted a limited toxicity due to Dox. This result was rationalized in terms of low drug loading in the nanoparticles, which is consistent with the difficult functionalization of the inner surface of the MSNs.

### 3.2. MSNs Containing a Drug inside the Pores and a Second Drug outside as Stopper

An alternate design of DDSs, which avoids the functionalization of the pores, consists of the use of one of the drugs as a stopper. In this strategy, one of the drugs is loaded inside the pores and the second drug is attached to the nanoparticle at the outlet of the pores by a stimulus-sensitive linker.

**Figure 4:**
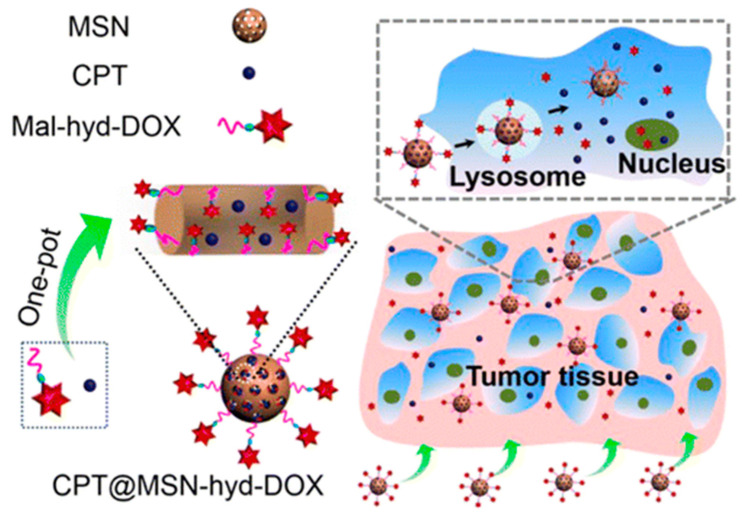
pH-responsive drug delivery system (DDS) for the dual delivery of Dox and camptothecin (CPT). Reproduced from reference [37] https://doi.org/10.1021/am402082d. Copyright 2013 American Chemical Society.

The group of Zhang reported in 2013 the preparation of a pH-responsive DDS, in which camptothecin (CPT) was physically loaded into MSNs and a chemically modified Dox with an acid-labile linker (CPT@MSN-hyd-Dox) was bonded to the surface of the MSNs. The linker was a difunctional chain containing a hydrazide and a maleimide moieties (Figure 4) [37]. The latter group reacted with thiols present onto the surface of the MSN. Therefore, this Dox derivative (Mal-hyd-Dox) was prepared in a four-step synthesis. Once in hand, this compound was reacted with the MSNs. At pH’s found in the lyso/endosomes of tumor cells (pH~5), the acidic hydrolysis of labile hydrazone bonds produces a burst release of Dox. At the same time, CPT was gradually freed from the pores of the nanoparticle. The cytotoxicity of the nanoparticles was assessed. According to the study, the combination of these two drugs led to a strong synergistic effect.

**Figure 5:**
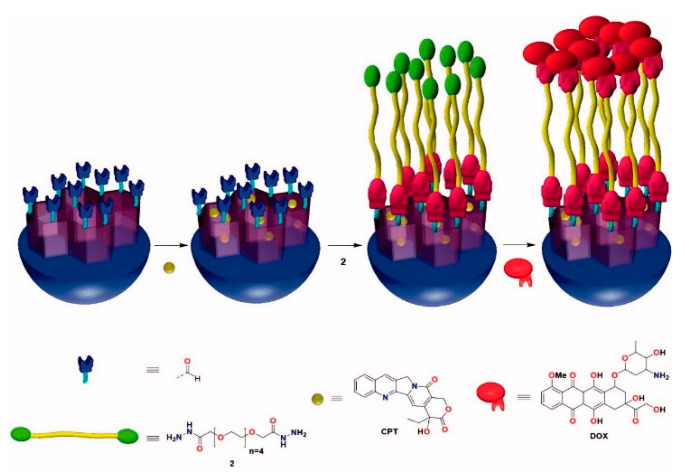
Preparation of a DDS for the delivery of Dox and CPT without derivatization of the drugs. Reproduced from reference [38] https://doi.org/10.1080/10717544.2018.1472678. Copyright 2018 Taylor & Francis Group.

A simple preparation of the DDS is a critical requirement to lead a DDS into clinics. Therefore, ideally, it should be avoided any derivatization of the drugs. According to this principle, in 2018 was reported a nano-vehicle for the delivery of Dox and CPT, which was prepared directly from the free drugs without the need of any derivatization step [38,39]. To do so, CPT was loaded inside the pores of the nanoparticle and Dox was covalently attached to the surface of regioselectively aldehyde-functionalized MSNs through a dihydrazide–polyethylene glycol chain (**2**) in a one-pot process (Figure 5). Interestingly, Dox and the linker act as an extremely efficient pH-sensitive gatekeeper since at physiological conditions the release of the drugs was negligible. By contrast, at acidic pH a burst release of Dox and a gradual release of CPT take place.

**Figure 6:**
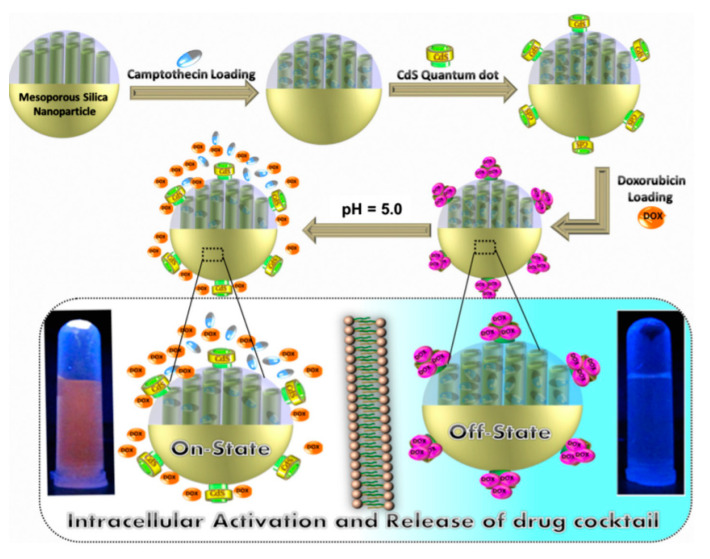
Schematic illustration of synthetic and operational mechanism of a co-delivery system of Dox and CPT based on CdS quantum dot gated MSNs. Reproduced from reference [40] https://doi.org/10.1016/j.jcis.2014.07.024. Copyright 2014 Elsevier.

The design of the stopper is crucial in preventing a premature leakage of the content of the pores. Zhu and collaborators suggested the use of CdS quantum dots. In this DDS, firstly CPT was loaded into the pores, then, CdS quantum dots were used to block the outlet of the MSNs (Figure 6) [40]. In the final step of the synthesis, Dox was anchored by absorption onto the surface of CdS. In response to a mildly acidic lysosomal environment Dox and CPT were simultaneously released. A similar nano-composite was reported in 2015 using ZnO quantum dots to seal the pores of hollow silica nanoparticles [41].

**Figure 7:**
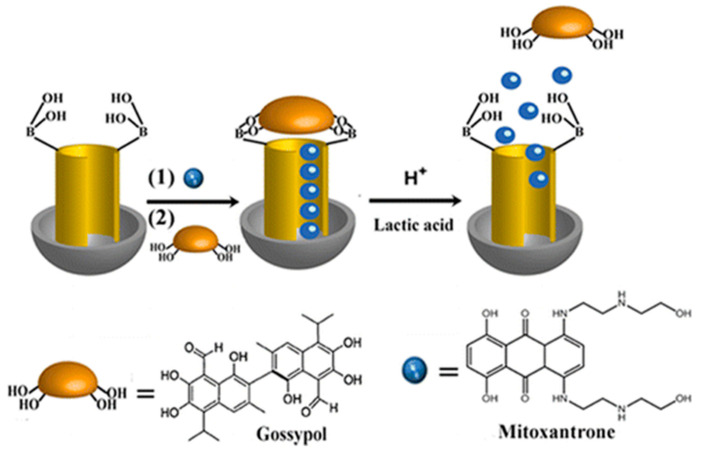
Gossypol-capped mitoxantrone-loaded mesoporous SiO_2_ nanoparticle’s system. Reproduced from reference [42] https://pubs.acs.org/doi/10.1021/acsami.6b03865. Copyright 2016 American Chemical Society.

The presence of functional groups in the structure of drugs can serve as inspiration for the design of smart gatekeepers. In 2016, Willner and co-workers envisioned a novel DDS taking advantage of the diphenolic symmetric structure of the anticancer drug gossypol [42]. First, MSNs functionalized with boronic acid moieties, which are known to react smoothly with vicinal cis-diols, were prepared. Then, the drug mitoxantrone was loaded into the voids of the MSNs, and the pores were capped with gossypol (Figure 7). Under mild acidic conditions and the presence of moderate concentrations of lactic acid, conditions usually found in cancer cells, the capping units unlocked the pores. This was attributed to the acidic hydrolysis of the boronate ester groups and to the cooperative separation of the boronate ester bridges by the lactate ligand. The cytotoxic effect of the combination gossypol-mitoxantrone was examined with MCF-10A breast cells and MDA-MB-231 breast cancer cells.

The amount of drug loaded within the pores of MSNs is limited, since a considerable volume of the particle is occupied by the silica matrix. As mentioned before, HMSNs are porous materials similar to MSNs but formed only by a thin, porous shell. Hence, the internal hole can be loaded with larger amounts of drugs. In 2017, Guo and collaborators prepared a co-delivery system based on HMSNs for the delivery of Dox and *cis*-7-(3-aminomethyl-cyclobutyl)-5-(3-benzyloxy-phenyl)-7*H*-pyrrolo[2,3-d]-pyrimidin-4-ylamine (NVP)-AEW541, an inhibitor of insulin-like growth factor receptor (IGF-1R) [43]. The DDS was prepared by treatment of HMSNs with *N*-[(3-trimethoxysily)propy]ethylenedi-amine triacetic acid trisodium to modify the surface of the nanoparticle with carboxylic acid groups. Then, Dox and NVP were loaded. The encapsulation efficiency of Dox and NVP were found to be 37% and 44%, respectively. The release of each drug was determined at different pH conditions (pH = 7.4, 6.5, 5.5, and 4.5). The results showed gradual smooth release of both drugs when the media becomes more acidic. The performance of the DDS was assessed with CD117^+^ CD44^+^ A2780 ovarian cancer stem-like cells showing an apoptosis rate almost three times higher than those of the free drugs group.

**Figure 8:**
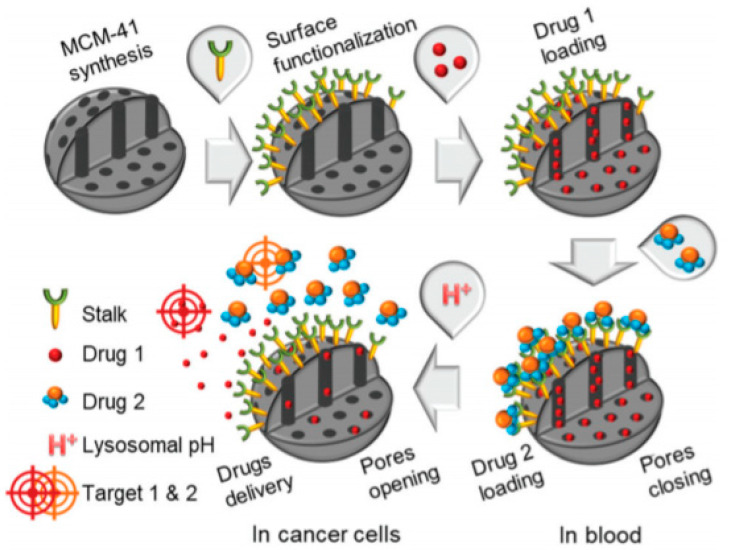
Conceptual combinatorial drug delivery MSN nanocarriers with Drug 1 (CPT) encapsulated inside the pores and Drug 2 (5-FU derivative) as the capping agent. Reproduced from reference [44] https://doi.org/10.1039/C9TB02225B with permission from The Royal Society of Chemistry.

In the design of complex systems, a supramolecular approach for the construction of the cap can be very useful. In 2020, Carcel and co-workers prepared MSNs functionalized with a specific organic ligand, which is able to recognize a complementary drug [44]. This supramolecular complex serves as a cap to block the pores of the nanoparticles (Figure 8). In particular, this ligand (stalk) was engineered to bind a trimer derivative of 5-fluorouracil (drug 2) [45]. A careful study of this system revealed that a negligible release of the drugs occurred at neutral conditions (pH = 7.4), thus avoiding toxic side effects. In contrast, within the pH found in endolysosomal compartments (pH~5.5) the hydrogen bonding of complex is destabilized, causing the concomitant release of the load (CPT, drug 1) and the cap (drug 2).

### 3.3. DDS Containing a Drug inside the Pores Using CD as Valve

Cyclodextrins have been captured the attention of chemists for decades. Their special topology and functionality make these macrocycles very useful building blocks in supramolecular chemistry and, in particular, the preparation of smart systems. CD are nontoxic oligosaccharides, consisting of a macrocyclic ring of glucose subunits joined by α-1,4 glycosidic bonds. The inner part of the ring is hydrophobic whereas the exterior surface is hydrophilic. These features permit the formation of host–guest complexes with hydrophobic compounds. In addition, the presence of hydroxyl groups at the rims of the rings, opens the door to further functionalization.

**Figure 9:**
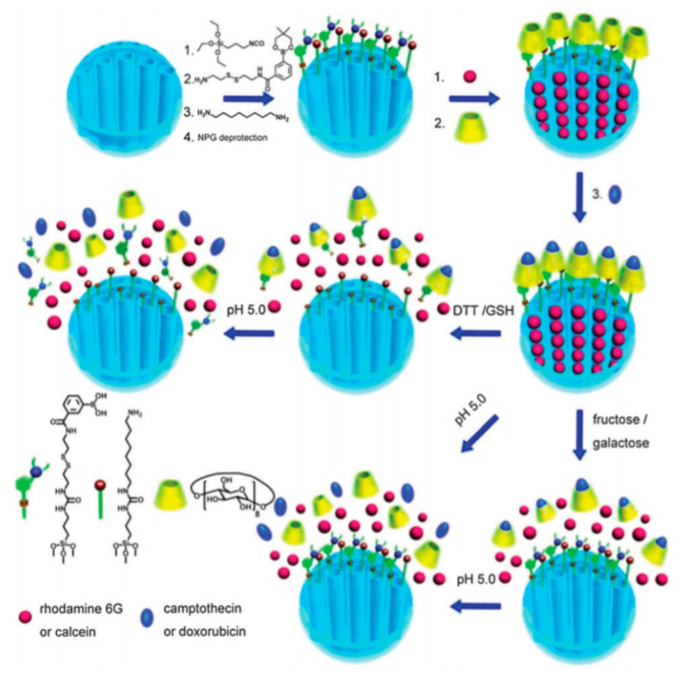
Preparation of γ-cyclodextrins (CD)-gated MSN vehicles functionalized with disulfide-linked carbamoylphenylboronic acid groups and amines and illustration of the cascade release of two drugs. Reproduced from reference [46] https://doi.org/10.1039/C5CC00585J with permission from The Royal Society of Chemistry.

In 2015, Du and collaborators presented a DDS consisting of a MSN decorated with γ-CD rings [46]. γ-CD were attached to the outer surface of the particles by means of disulfide-linked carbamoylphenylboronic acid moieties (Figure 9). The aim of such functionalization was the blocking of the pores outlets. Three release pathways were envisioned: Hydrolysis of the boronate esters under acid conditions, cleavage of the disulfide bond by reductive agents such as dithiothreitol (DTT) or GSH and competitive binding of monosaccharides to the boronate moieties, which would liberate the γ-CD macrocycles. Remarkably, the role of the γ-CD is not only to act as a stopper, but thanks to its inner cavity to accommodate a hydrophobic drug. Furthermore, the external hydrophilic surface of the γ-CD would improve the biocompatibility of the MSNs. In this study, Dox was entrapped in the MSN pores and CPT was encapsulated within the γ-CD cavities.

**Figure 10:**
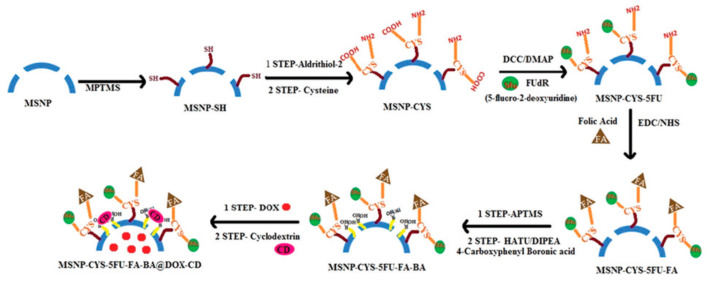
Preparation of MCM-41 for the delivery of Dox and 5-fluoro-2-deoxyuridine decorated with folic acid (FA). Reproduced from reference [47] https://doi.org/10.1039/C9TB02628B with permission from The Royal Society of Chemistry.

The improvement of the selective accumulation of the DDS in the tissue of interest is of the utmost importance for an efficient treatment. To this end the particles have been conjugated with a variety of ligands able to recognize over-expressed receptors in cancer cells. Thus, in 2020, Manna and co-workers prepared a complex triple stimuli-responsive drug delivery platform for the delivery of Dox and 5-fluoro-2-deoxyuridine (5FU) which included folic acid (FA) as a ligand to target cancer cells (Figure 10) [47]. The nano-vehicle was synthetized starting from MSN-SH, which were functionalized with cysteine via disulfide bonds. The carboxylic acid groups were activated by reaction with *N*,*N*’-dicyclohexylcarbodiimide (DCC) in the presence of 4-dimethylaminopyridine (DMAP), which formed ester bonds with ribose alcohol groups present in 5FU. The remaining amino groups were used to attach FA following the same procedure. Subsequently, the external surface of the nanoparticles was decorated with 4-carboxyphenylboronic acid. Finally, Dox was entrapped within the silica porous and these were sealed with CDs via molecular interactions between the boronic acid and vicinal diols in the CD. According to the authors, the construct was very effective against Dalton’s lymphoma (DL) and other types of cancer cells, such as breast adenocarcinoma (MCF-7) and human erythroleukemia (K 562).

**Figure 11:**
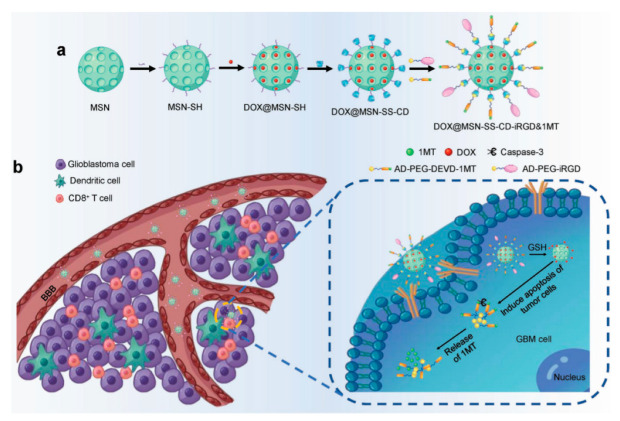
(**a**) Preparation of Dox@MSN-SS-CD-iRGD&1-methyltryptophan (1MT). (**b**) Illustration of the release mechanism. Reproduced from reference [48] https://doi.org/10.1002/adfm.201800025. Copyright 2018 Wiley-VCH verlag GmbH & Co. KgaA, Weinhem.

An analogous approach was used by He, Liu and co-workers for the dual delivery of Dox and the immune checkpoint inhibitor 1-methyltryptophan (1MT) [48]. In this instance, MSN-SS-alkyne were prepared and reacted with azide-CD. The CD macrocycle was utilized as a linker and stopper to decorate the MSNs surface with 1-methyltryptophan and iRGD, a peptide that can penetrate to the tumor tissue and target the tumor cells (Figure 11). To do so, both groups (1-methyltryptophan and iRGD) were derivatized with adamantane, which allows the host-guest interaction that tether these molecules to the MSNs. The main advantage of this supramolecular linkage is the simple introduction of complex molecules to the final system. In addition, 1MT was attached to the adamantane molecule by a peptidic linker (Asp-Glu-Val-Asp; DEVD) sensitive to the presence of caspase-3. This delivery platform was tested to assess the application of antitumor immunity techniques in the treatment of glioblastoma.

**Figure 12:**
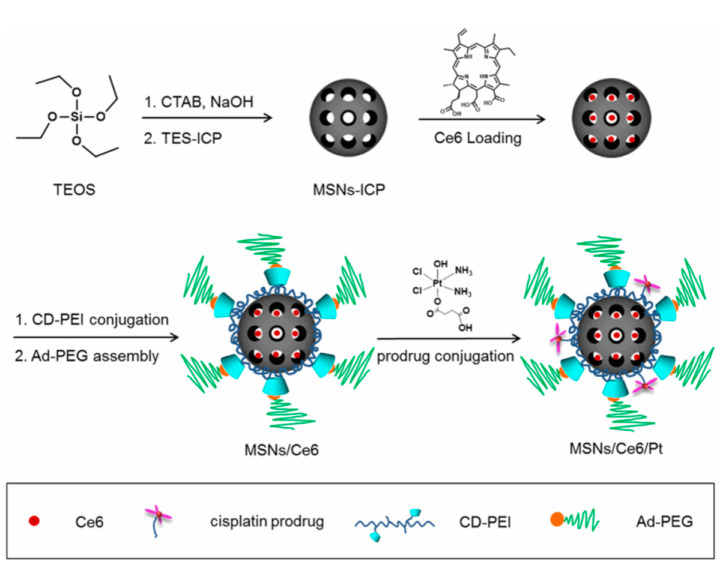
Fabrication of a DDS containing a cisplatin prodrug and photosensitizer Ce6. Reproduced from reference [49] https://doi.org/10.1021/acsami.6b03881. Copyright 2016 American Chemical Society.

As mentioned, the CD-adamantane complex can be very handy as a supramolecular linker. In some cases, the use of this strategy probably surpasses in efficiency the well-known “click chemistry”. For instance, allows the easy pegylation of nanoparticles (NP) surface, which endow the DDSs with stability and biocompatibility. Usually, pegylation is carried out by reaction of NPs with polyethylene glycol (PEG) derivatives with the aid of coupling reagents. However, the host-guest chemistry can offer a novel solution. In 2016, Mao and co-workers reported the preparation of a DDS based on MSNs for cisplatin and chlorin Ce6 delivery, which takes advantage of this technique (Figure 12) [49]. The preparation of such DDS entails five steps. First, 3-isocyanatopropyl-modified MSNs were prepared by grafting of MCM-41, then, the photosensitizer was loaded within the pores of MSNs. In the next step the particles were capped with a β-cyclodextrin-grafted polyethylenimine (CD-PEI). Then, the resulting particles were decorated with the PEG-Adamantane chain to accomplish the pegylation of the DDS. Finally, cisplatin bearing a carboxylic acid group was reacted with the amino groups of CD-PEI using the 1-ethyl-3-(3-dimethylaminopropyl)carbodiimide (EDC) mediated coupling. This DDS was used to explore the beneficial effects of PDT to circumvent cisplatin resistance.

### 3.4. DDSs Loaded with One Drug and Coated with a Polymer

One of the main requirements for a DDS is to be stable in blood without releasing the drug prematurely. This feature can be introduced into the design of the nano-vehicle by using polymers [50]. Furthermore, polymers are well known materials to prepare stealth nanoparticles, which allows evading immune recognition to enhance its circulation time in vivo. Examples of polymers used in drug delivery applications are dextran (Dex), chitosan (CH) [51], polyvinyl alcohol (PVA), and PEG.

**Figure 13:**
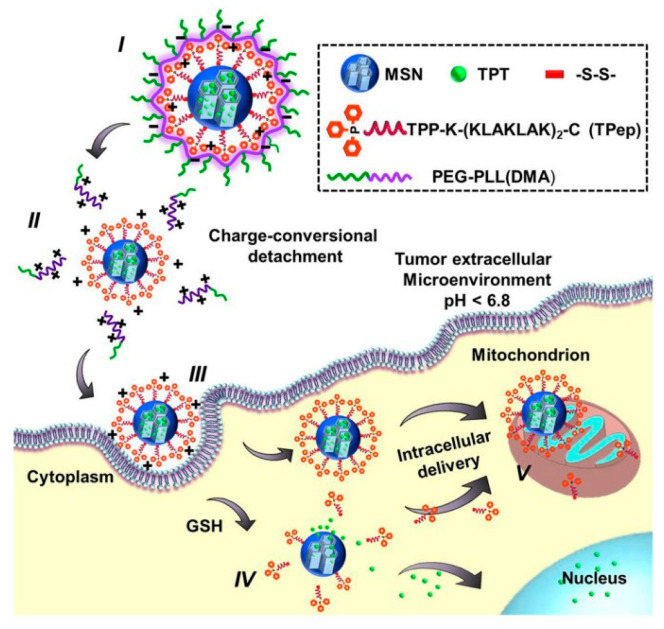
MMS coated with a charge reversal polyanion for the delivery of a mitochondria-targeted therapeutic agent (Tpep) containing triphenylphosphonium (TPP) and antibiotic peptide (KLAKLAK)_2_ and topotecan (TPT). Reproduced from reference [52] https://doi.org/10.1038/srep06064. Copyright 2014 McMillan Publishers.

In 2014, Zhang’s group reported the preparation of an enveloped nano-device for delivery of topotecan (TPT) and an antibiotic peptide (KLAKLAK)_2_ [52]. This DDS possesses a complex architecture. First, TPT was loaded inside the MSNs. Then, the surface of the nanoparticles was decorated with a mitochondria-targeted therapeutic agent (Tpep) containing triphenylphosphonium (TPP) and antibiotic peptide (KLAKLAK)_2_ via disulfide linkages (Figure 13). Finally, these MSNs were coated with a charge reversal polyanion poly(ethylene glycol)-blocked-2,3-dimethylmaleic anhydride-modified poly(L-lysine) (PEG-PLL(DMA)) via electrostatic interaction (I). The authors claim that the outer polymer layer can be removed at acidic tumor microenvironment due to the degradation of DMA blocks and the cellular uptake was significantly enhanced by the formation of cationic nanoparticles (II).

**Figure 14:**
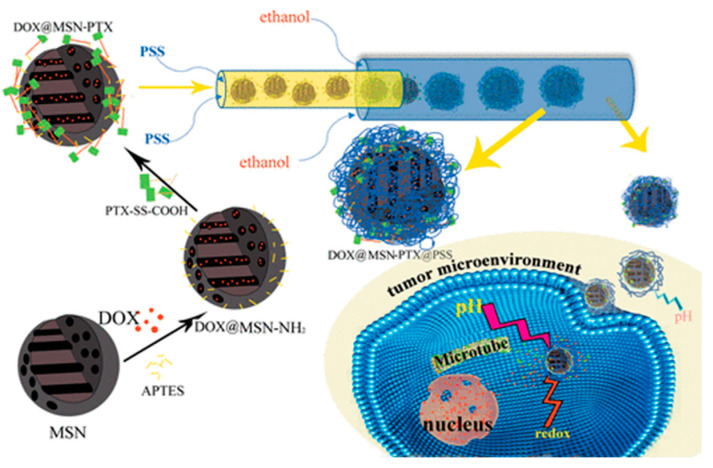
Polystyrenesulfonate coated MSN for the co-delivery of Dox/ paclitaxel (PTX). Reproduced from reference [53] https://pubs.acs.org/doi/10.1021/acsabm.9b01111. Copyright 2020 American Chemical Society.

In 2020, polystyrenesulfonate (PSS) coated MSNs were engineered for the dual delivery of Dox/paclitaxel (PTX) [53]. In this DDS, PTX was covalently attached to the surface of Dox loaded MSNs via a linker with disulfide bond (Figure 14). According to the authors, the PTX’s loading degree can be easily tuned to achieve the optimum drug loading ratio to Dox, to maximize the synergistical effect. In the final step, the loaded nanoparticles were electrostatically coated with PSS using microfluidics. The resulting DDS was redox and pH-responsive since the electrostatic interaction between the MSN and the polymer would be neutralized at pH 5. The cytotoxic profile of this DDS was tested against cancer cell BT549 and healthy breast cell MCF-10A.

## 4. Nano-systems Based on MSNs for the Delivery of Combination of Drugs and Nucleic Acids

After intravenous injection, naked RNA is prone to degradation by ribonucleases. Therefore, it must be protected by encapsulation to assure a correct delivery to the cells [54]. In the last decade, many studies have demonstrated that MSNs are efficient vehicles to protect polynucleotides from enzymatic activity and lowering the immunogenicity of RNA. Besides, the versatile chemistry of MSNs allows the functionalization of particles to improve their internalization and endosomal escape [55]. Three main strategies have been developed for the dual transport of drugs and RNA: DDSs with the outer surface of the particle decorated with RNA, MSNs coated by a polycationic polymer, which acts as gate-keeper and nanoparticles that carry the RNA inside the pores. Out of these approaches, the best studied system is the one using MSNs coated with a cationic polymer. This polymer protects the fragile RNA from degradation and prevents any premature leakage of the cargo inside the pores. In turn, four variants of this class of DDS have been reported: DDSs with polymers attached by electrostatic interaction, DDSs with the polycation attached covalently, MSNs with polycations attached with a cleavable linker, and nanoparticles attached to a polycation decorated with a targeting ligand.

### 4.1. DDSs with RNA Attached Covalently onto the Surface of the MSNs

A simple approach to engineer NPs able to deliver a drug and siRNA consists in the covalent attachment of one of the ends of the RNA strand to the surface of the MSN and storing the drug inside the porous interior. Under the proper conditions, RNA will be liberated allowing the concomitant release of the drug from the pores. Following this design, in 2011, Minko and co-workers presented a DDS for the delivery inside cancer cells of Dox and cisplatin combined with two types of siRNA targeted to MRP1 and B-cell lymphoma 2 (Bcl-2) mRNA for suppression of cellular resistance in non-small cell lung carcinoma, respectively [56]. The nano-vehicle was built using thiol modified MSNs, which were activated by treatment with Aldrithiol™ to produce pyridyldithiol reactive groups. Once Dox or cisplatin were loaded within the pores, the nanoparticle was treated with the corresponding 5′ thiol-siRNA. Furthermore, to endow selective tumor-targeting the DDS was conjugated to luteinizing hormone-releasing hormone (LHRH). To this end, heterobifunctional HS-PEG-COOH was used as linker, and LHRH was attached via amide bond.

**Figure 15:**
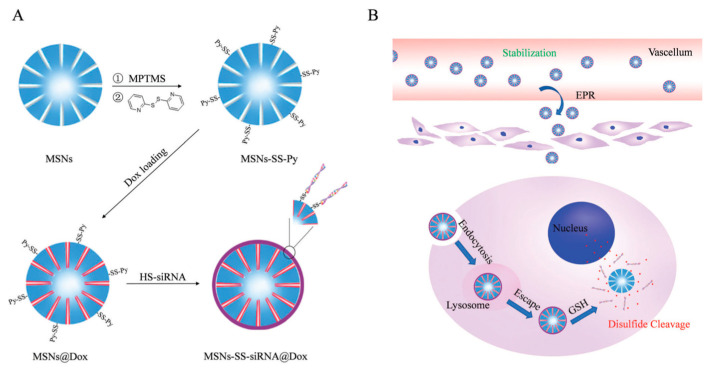
Schematic diagram showing (**A**) the synthetic procedure for MSNs-SS-siRNA@Dox and (**B**) the extracellular and intracellular trafficking of MSNs-SS-siRNA@Dox to cancer cells. Reproduced from reference [57] https://doi.org/10.1039/C7TB00613F with permission from The Royal Society of Chemistry.

A similar DDS (MSN-SS-SiRNA@Dox) was presented in 2017 by Liu and col [57]. Analogously, the release of siRNA and Dox was triggered by exposing the system to GSH. According to this study, the particles successfully escaped from lysosomal degradation and inhibited the expression of Bcl-2 protein. Biological in vitro and in vivo experiments were carried out showing that the system exhibited satisfactory cytotoxicity by inducing apoptosis in MCF-7 cells (Figure 15).

### 4.2. MSNs Coated by a Polycation

As mentioned, the exposure of RNA and DNA to biological fluids provoke a rapid degradation of their chemical structure. To avoid or slow down this process it has been proposed the use of cationic polymers able to encapsulate RNA/DNA. The ionic interaction between the negative charged nucleic acid and the polymers assemble a protective complex that ensures the integrity of the cargo. Depending on the structural features of this cationic polymer the resulting system can be categorized into four design types: DDS with the polymer attached by electrostatic forces, MSNs with polymers linked covalently, MSNs coated with polymers tethered by cleavable bonds, and NPs decorated with polymers conjugated with targeting ligands.

#### 4.2.1. Polymer Attached to the NP by Electrostatic Interactions

In 2010, Zink and co-workers presented a simple yet effective DDS based on MSNs coated with polyethyleneimine (PEI) (1.8–2.5 kDa) to deliver the chemotherapeutic agent Dox as well as P-glycoprotein (P-gp) siRNA to a drug-resistant cancer cell line (KB-V1 cells) [58]. The preparation of such system entails the modification of the MSNs surface with trihydroxysiylpropyl methylphosphonate. The phosphonate coating plays two roles: Binds Dox via a proton-sensitive mechanism, enhancing the loading of the drug, and provides a negatively charged surface for binding the cationic polymer. In turn, the cationic polymer complexes the siRNA protecting the fragile cargo from degradation. Furthermore, the complex formed by siRNA and PEI acts as pH-sensitive cap to avoid the premature leakage of the cargo. According to the authors, the dual delivery of Dox and siRNA was capable of increasing drug concentration in cells to levels exceeding that of free Dox in the absence of siRNA co-delivery.

**Figure 16:**
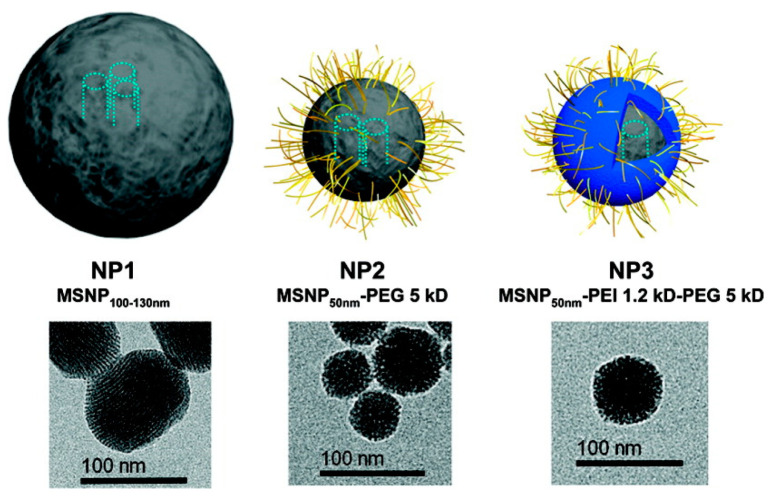
MSNs coated with a polyethyleneimine (PEI)-polyethylene glycol (PEG) co-polymer. Reproduced from reference [58] https://pubs.acs.org/doi/10.1021/nn100690m. Copyright 2010 American Chemical Society.

The following year the same group reported a second generation of their DDS. It was found that the use of smaller MSNs (50 nm) and a coating of PEI-PEG co-polymer (PEI (1.2 kD) and PEG (5 kD)) produced a great impact in the biodistribution of the DDS. In particular, the passive delivery was enhanced and a reduction of the particle opsonization was accomplished (Figure 16).

**Figure 17:**
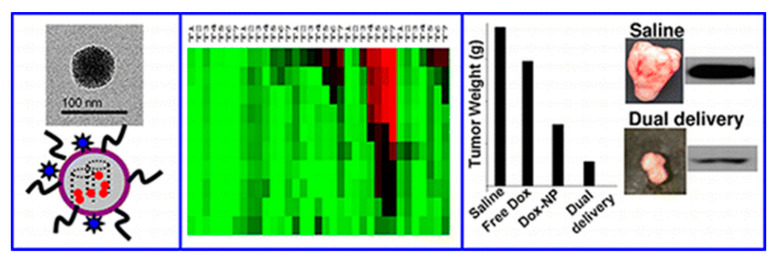
Determination of the optimal Dox/siRNA combination to overcome drug resistance in breast adenocarcinoma (MCF-7)/MDR cells. Heat map display of the HTS cytotoxicity assays were carried out using the MTS reagent in 384-well plates. The data were quantitatively expressed using MeV software to generate heat maps, in which each of the rows and columns represent dose range and exposure times (T1–T7), respectively. A green color indicates lack of cytotoxicity, while red indicates a significant killing effect. Reproduced from reference [59] https://pubs.acs.org/doi/10.1021/nn3044066. Copyright 2013 American chemical society.

In 2013, an optimized version of the later DDS was studied for the delivery of a series of siRNAs and Dox. In this instance, 50 nm MSNs were coated with a PEI (1.8 kD)-PEG (5 kD) co-polymer [59]. These nanoparticles were tested in a high throughput screening assay to find the optimal siRNA/drug combination for overcoming Dox resistance in MCF-7/MDR cells. Six siRNA were selected to find the best combination for overcoming this resistance: P-gp, MRP1, ABCG2 (ABC drug efflux transporters), Bcl-2 (antiapoptotic protein), cMyc (oncogene involved in multidrug resistance), and PXR (expression of the regulator of drug-metabolizing enzyme). The study demonstrated that P-gp knockdown provided the best cell killing by Dox, while siRNAs targeting MRP1, ABCG2, Bcl-2, cMyc, or PXR had a lesser effect (Figure 17).

The versatility of the MSNs coated by PEI thanks to the ionic interaction with methylphosphonates have been proved by other researchers. In 2018, Wang, Xu and co-workers prepared a DDS based on this design capable to deliver MDR1-siRNA and Dox, simultaneously [60]. It was found that MDR1-siRNA was efficiently transfected into KBV cells in vitro resulting with a clear decrease gene expression of MDR1 and an apoptosis induction of the cells (24% after 48 h). In vitro studies demonstrated that the MSNP-PEI-Dox/MDR1-siRNA reduced the tumor size (82% decrease after 28 days posttreatment).

Although the methylphosphonate coating presents some advantages over the unfunctionalized surface of the MSNs, it must be point out that the silanol groups of the silica matrix endow the particles with an anionic character. Thus, in principle it is possible the coating of MSNs with PEI without any modification of the particle [61]. Accordingly, in 2018, Dilnawaz and co-workers reported the application of unfunctionalized MSNs for the co-delivery of anticancer drugs (docetaxel, etoposide, and carfilzomib) along with survivin siRNA for a comparative therapeutic study about their efficacy in lung cancer [62].

**Figure 18:**
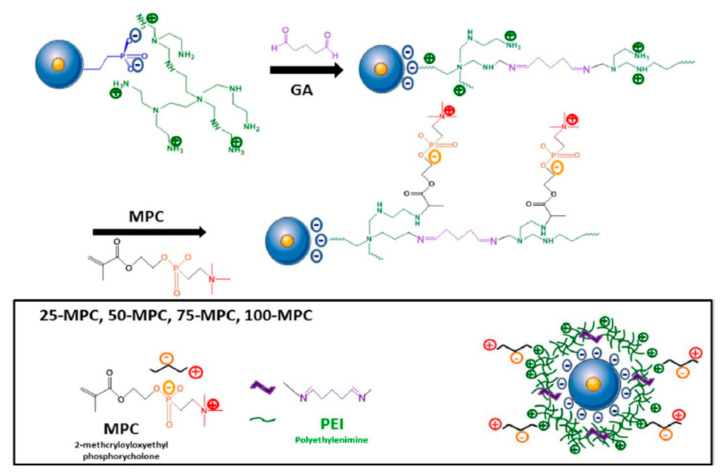
Preparation of zwitterionic PEI coated core-shell Fe_3_O_4_@SMSNs. Reproduced from reference [67] https://doi.org/10.1016/j.cej.2017.12.116. Copyright 2018 Elsevier.

The copolymerization of PEI with PEG helps to diminish the moderate toxicity of PEI [61,63]. Furthermore, it has been noted that the presence of PEG can be beneficial for the reduction of the enzymatic degradation of siRNA. Another advantage of pegylation is the reduction of the binding of blood proteins to the nanoparticles, which leads to opsonization and decreases blood circulation time. However, PEG coating presents some limitations such as the reduction of the uptake of the particles [64,65,66]. An effective alternative to pegylation is the introduction of zwitterionic polymers. An example of the application of such polymers for the preparation of a DDS with anti-fouling capacity was presented in 2018 by the groups of Vallet–Regí and Zink [67]. In this work, PEI coated core-shell Fe_3_O_4_@SiO_2_ MSNs were modified with a zwitterionic 2-methacryloyloxyethyl phosphorylcholine coating. The synthesis is quite straightforward. First, core-shell Fe_3_O_4_@SiO_2_ mesoporous nanoparticles were reacted with (2-diethylphosphatoethyl)triethoxysilane (DPTES) in order to introduce the phosphonate group on the surface of the nanoparticles. Then, a homogeneous suspension of the particles was sonicated in the presence of PEI. Subsequently, to this suspension a solution of glutaraldehyde was added. After 30 min, the resulting nanoparticles were treated with solutions of 2-methacryloyloxyethyl phosphorylcholine (MPC) during 24 h to introduce different MPC/DPTES ratios in the coating. The resulting DDSs were studied for the co-delivery into cancer cells of the anti-TWIST siRNA and daunorubicin (Figure 18).

#### 4.2.2. Polycation Attached Covalently to the NP

**Figure 19:**
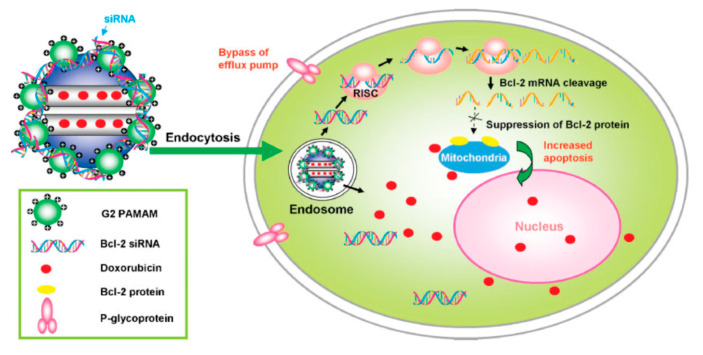
Schematic diagram of a co-delivery system based on MSNs to deliver Dox and B-cell lymphoma 2 (Bcl-2)-targeted siRNA simultaneously to A2780/AD human ovarian cancer cells. Reproduced from reference [68] https://doi.org/10.1002/smll.200900621. Copyright 2009 Wiley-VCH verlag GmbH & Co. KgaA, Weinhem.

An alternative method to anchor the polycationic polymer is by formation of a covalent bonding. An early example of this approach was reported in 2009 by Minko, He, and collaborators [68]. In this work, a MSN-based nano-vehicle for the co-delivery of Bcl-2 targeting siRNA and Dox to A2780/AD human ovarian cancer cells was described. As a polycationic element a generation 2 amine-terminated polyamidoamine (G2PAMAM) was chosen (Figure 19). Briefly, the surface of the MSN was modified by treatment with 3-isocyanatopropyltriethoxysilane (ICP) to introduce the isocyanatopropyl group. Then, the pores of the resulting particles (MSN-ICP) were loaded with Dox. Finally, the exposure of the particles (MSN-ICP) to the amines of dendrimer formed a urea linkage between the MSNs and G2PAMAM. The loading efficiency of Dox determined by UV-vis spectroscopy was found to be approximately 40%. The as-synthesized particles where complexed with the corresponding siRNA. The efficiency of the encapsulation was determined from an agarose gel electrophoresis experiment. This analysis indicated that all siRNAs formed stable complex with MSN-Dox-G2 at N/P = 1, where N represents the number of charged primary amine groups of the G2 dendrimer on the MSNs and P is negatively charged phosphate groups from siRNAs.

One year later, Oupicky and co-workers replaced the dendrimer as polycation and coated the MSNs with PEG-poly(2-(dimethylamino)ethylmethacrylate) (PDMAEMA) and PEG-poly(2-(diethylamino)ethyl-methacrylate) (PDEAEMA) copolymers [69]. The aim of the study was assessing the effect of chloroquine (CQ), a known lysosomotropic agent, on the transfection and silencing activity of DNA and siRNA. To prepare the nano-container, firstly, aminated MSNs were treated with PEG maleimide. Once in hand, the MSN-PEG were reacted with PDEAMA-COOH or PDMAEMA-COOH previously activated with *N*-hydroxysuccinimide in the presence of DCC. Then, the particles were loaded with the free base of CQ. Finally, complexes of the nanoparticles with gWIZ-Luc plasmid DNA, anti-luciferase siRNA, anti-GAPDH siRNA using a 0.02 M sodium acetate buffer were prepared. The authors concluded that the co-delivery of CQ and the nucleic acids led to a significantly increased transfection and silencing activity of the complexes compared with MSNs not loaded with CQ.

**Figure 20:**
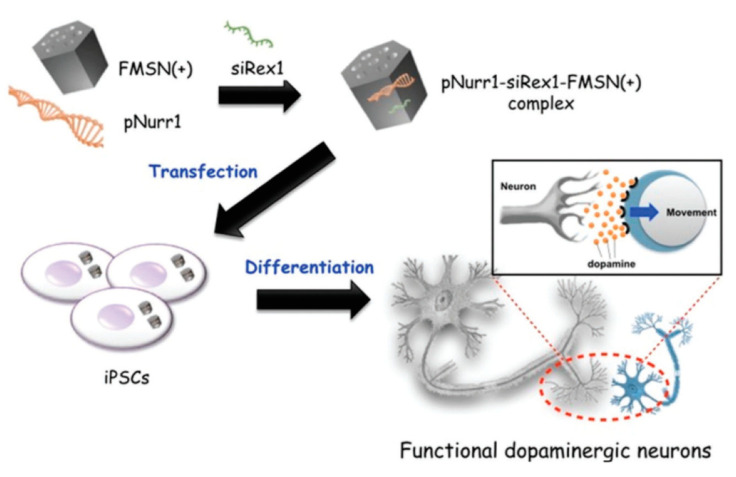
Nurr1 plasmid DNA and Rex1 siRNA complexed with the fluorescein isothiocyanate (FITC)-conjugated cationic MSN (FMSN(+)) by adsorption. Reproduced from reference [70] https://doi.org/10.1039/C7TB00351J with permission from The Royal Society of Chemistry.

When the protective coating for siRNA is considered not necessary, cationic MSNs without a polymer can be simpler to prepare. In 2017, Chiou, Mou, and collaborators described the use MSNs functionalized with trimethylammonium groups for the co-delivery of Nurr1 plasmid DNA (pNurr1) and Rex1 siRNA (siRex1) into iPSCs to achieve dopaminergic neuron differentiation [70]. Such particles were easily prepared by exposure of the MSNs to *N*-trimethoxysilylpropyl-*N*,*N*,*N*-trimethylammonium (TMA) in ethanol at reflux for 12 h. The dopaminergic neuron-related protein level was identified using immunofluorescence. The results obtained allow the authors to claim that the co-delivery enhanced the Nurr1 gene expression three-fold, compared to the delivery of the plasmid pNurr1 alone (Figure 20).

#### 4.2.3. Polycation Attached with a Cleavable Linker

The extraordinary development of smarts DDSs based on MSNs has provide a wide variety of capping systems to control the delivery [29,30]. As a consequence, these sophisticated systems allow the fine tuning of the release of multiple drugs and open the door to precise treatments. To gain selectivity in the release of the cargo, a common strategy relies on the use of stimuli-sensitive linkages. These cleavable linkers are dissociated under specific conditions of pH, high concentration of GSH, or exposure to light to name some triggering the liberation of the drug.

**Figure 21:**
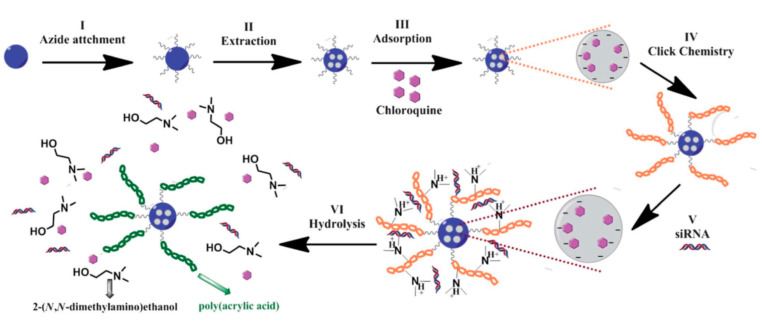
Synthetic scheme for the preparation of poly(2-dimethyl-aminoethyl acrylate) (PDMAEA)- large pore mesoporous silica nanoparticles (LPMSN) composites. Reproduced from reference [71] https://doi.org/10.1039/C3TB21015D with permission from The Royal Society of Chemistry.

An example of DDS endowed with dual release triggered by acidic pH’s was presented in 2014 by Monterio, Yu, and collaborators [71] (Figure 21). This group of researchers reported a DDS based on large pore mesoporous silica nanoparticles (LPMSN) as gene carrier, which were coated with a degradable polymer poly(2-dimethyl-aminoethyl acrylate) (PDMAEA). Under mild acidic conditions, PDMAEA underwent a self-catalyzed hydrolysis to form the non-toxic polymer poly(acrylic acid) (PAA) and 2-(*N*,*N*-dimethylaminoethyl)ethanol as by-products. Concomitant to this hydrolysis, the cargo of the pores (CQ) and siRNA were released. The synthesis of this DDS entails the functionalization of LPMSN with azido groups by grafting with 2-azido-*N*-(3-(triethoxy silyl)propyl)propenamide. After extraction of the surfactant and CQ loading, the nanoparticles were coated with the polymer using “click chemistry”. Thus, azido-modified particles were treated with poly(2-dimethylaminoethyl acrylate) functionalized with terminal acetylene moieties (PDMAEA) in the presence of CuBr as catalyst and *N*,*N*,*N*′,*N*′′,*N*′′-pentamethyldiethylenetriamine (PMDETA) as ligand in toluene at room temperature for 24 h. The as-synthetized nanoparticles were complexed with oligo DNA at two different nitrogen to phosphorous ratios (N/P) (N/P = 1 and 10). As anticipated, the co-delivery of CQ enhanced the endosomal escape of the DNA and, consequently, the transfection efficiency.

In 2017, Tang and co-workers prepared a pH-responsive co-delivery system for doxorubicin and survivin short-hairpin RNA (shRNA) based on aminated MSNs which formed a Schiff-base with 2-formylimidazole [72]. According to the authors, the role of the imidazole ring is threefold since improves loading capacity of the nucleic acids, enhances cellular uptake thanks to the electrostatic interaction with the membrane of cancer cells and facilitates the endosome’s rupture by the “proton sponge” mechanism [73] improving the transfection. The preparation of such DDS is quite simple and the mechanism of release of the cargo is the hydrolysis of the imine bonded to the nanoparticle. Hence, under acidic conditions the release of both Dox and siRNA takes place. The efficiency of the system was tested in vitro (Human hepatoma cell line QGY-7703) and in vivo.

**Figure 22:**
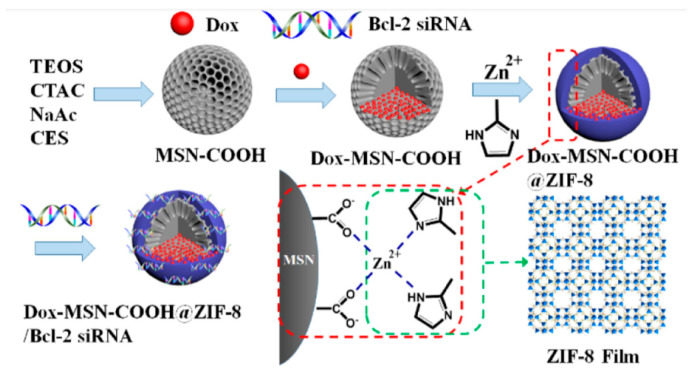
Carboxylated silica nanoparticles synthesized in situ capped with zeolite imidazole framework 8. Reproduced from reference [74] https://doi.org/10.1021/acsami.8b13393. Copyright 2018 American Chemical Society.

Another feature of interest of the imidazole is its ability to act as metal ligand. In 2018, Chu and co-workers, prepared an innovative DDS based on a zeolitic imidazole framework-8 (ZIF-8) film with a few nanometer thickness built using carboxylated MSNs (Figure 22) [74]. The nanoparticles were used as reservoir of Dox and the capping agent consisted in a Zn complex, which in turn, gave rise to the ZIF-8 film. This complex was formed by participation of the carboxylate groups from the MSNs surface and 2-methyl imidazole. The positive surface of the film allowed efficient loading of siRNA via electrostatic interactions and protect siRNA from nuclease degradation. The film can decompose under acidic conditions, which trigger the intracellular release of siRNAs and Dox. According to the biological tests, the combined action of the siRNA and Dox led to a significantly enhanced chemotherapeutic efficacy for multidrug-resistant cancer cells MCF-7/ADR and SKOV-3/ADR.

**Figure 23:**
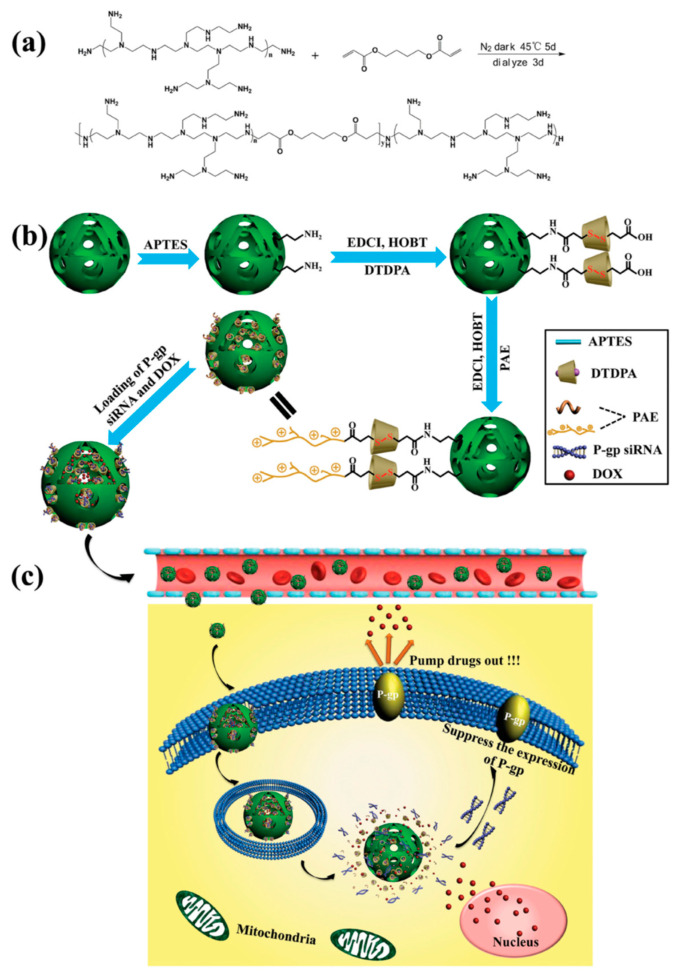
(**a**) Synthesis of the polyaminoester (PAE) (**b**) Construction of P-glycoprotein (P-gp) modulator siRNA and Dox co-loaded HMONs-ss-PAE system. (**c**) Proposed mechanism for the drug release inside the cells. Reproduced from reference [75] https://doi.org/10.1002/adma.201505524. Copyright 2016 Wiley-VCH verlag GmbH & Co. KgaA, Weinhem.

It is well-known that the concentration of GSH inside cancerous cells is in the range of 2–8 mM, while in plasma is 1–2 μM. Capitalizing on this differential concentration of GSH, in 2016, Li, Shi, and co-workers devised a redox-responsive vehicle based on HMSNs to simultaneously deliver P-gp modulator siRNA and Dox to reverse the multidrug resistance (MDR) of breast cancer cells (Figure 23) [75]. The DDS was prepared in three steps. First, aminated HMSNs were coupled with 3,3′-dithiodipropionic acid through an amidation reaction. The diacid will serve as redox-sensitive linker between the nanoparticles and the polycation. In this nano-system, the polycation employed was a polyaminoester (PAE). This polymer was prepared via Michael addition reaction by treatment of PEI600 with 1,4-butanediol diacrylate (mole ratio: 1.5:1) at 45 °C for 5 days. Finally, HMONs-ss-PAE were obtained by the covalent conjugation between HMONs-SS-CO_2_H and PAE. According to the authors, this particular PAE is highly biocompatible and facilitates the intracellular uptake of the nanoparticles across the cell membrane and the endosomal escape via the “proton sponge” effect. In addition, the polymer degrades into small fragments because of the hydrolysable ester bonds leading to siRNA release. The last step of the DDS preparation entails the encapsulation of Dox and P-gp modulator siRNA into HMONs-SS-PAE. A similar approach was used in 2020 by Xiazeng, Luo, and co-workers for the delivery of CPT and Bcl-2 siRNA [76].

Premature leakage of the drug from the DDS is one major concern in drug delivery. The introduction of elements of supramolecular chemistry have provided new tools to minimize it. An example of that is the use of CDs as building blocks for the design of smart valves and linkages between nanoparticles and biomolecules. In 2014, it was reported the preparation of a redox-responsive vehicle based on cyclodextrin-gated mesoporous silica nanoparticles to simultaneously deliver Bcl-2 siRNA and Dox [77]. First, the surface of the nanoparticles was functionalized with adamantane (AD) units using a disulfide linker. Subsequently, Dox was loaded into the MSNs mesopores. Then, the voids were capped by formation of stable host–guest complex between the AD molecules and ethylenediamine-modified β-cyclodextrin. Finally, the amino functionalized CD were used to complex siRNA via electrostatic interactions. In vitro and in vivo experiments demonstrate an enhancement of the cytotoxicity caused by simultaneous delivery of Bcl-2 siRNA and Dox in HeLa cells. Furthermore, a delivery experiment carried out in transgenic zebrafish larvae indicated that the delivery of Dox inhibits the development of choroid plexus in a dose-dependent manner.

**Figure 24:**
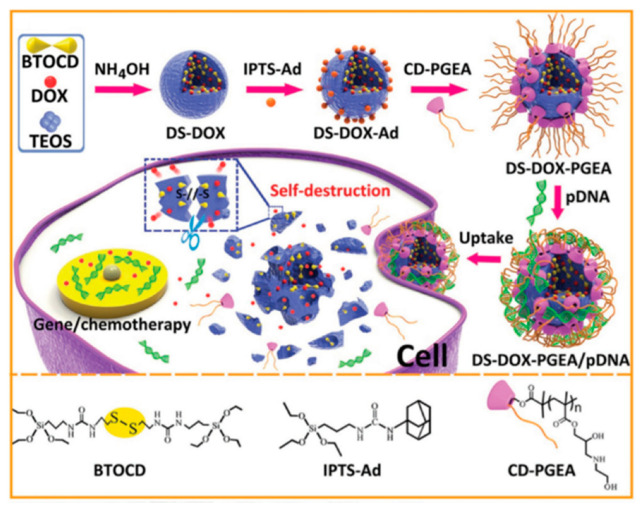
Schematic illustration of the preparation of DS-Dox-PGEA and the resultant stimuli-responsive drug/gene co-delivery process. Reproduced from reference [78] https://doi.org/10.1002/adfm.201606229. Copyright Wiley-VCH verlag GmbH & Co. KgaA, Weinhem.

The concept of attaching a polymeric coating for siRNA complexation using CD as linkers was applied again three years later by Zhao, Xu, and collaborators. In this case, instead of MSNs redox-responsive and self-destructive silica nanoparticles were used as inorganic scaffold [78]. The redox-responsive silica nanoparticles were synthesized by a facile one-pot method using TEOS and a disulphide-bridged disiloxane (BTOCD) as silica precursors. Dox was embedded into the as-synthesized particles. Subsequently, these nanoparticles were decorated with the adamantane moiety by grafting with the corresponding siloxane, IPTS-Ad. Finally, cationic polymer CD-PGEA, incorporating one β-cyclodextrin (β-CD) core and two ethanolamine-functionalized poly(glycidyl methacrylate) arms, was assembled onto the surface of the particles via the β-CD and Ad interaction. Once the polycation was attached, the nanoparticles were exposed to plasmid p53 to yield the final nano-vehicle (Figure 24).

**Figure 25:**
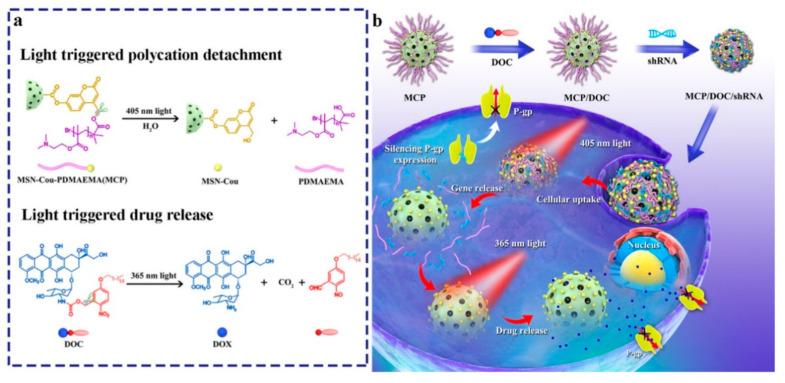
(**a**) Structure and photolysis of photoresponsive vehicle and DOX prodrug (DOC). (**b**) Schematic illustration of sequential release of short-hairpin RNA (shRNA) and DOX regulated by 405 and 365 nm light irradiations. Reproduced from reference [79] https://doi.org/10.1021/acsami.8b03823. Copyright 2018 American Chemical Society.

In addition to internal stimuli such as pH and elevated concentrations of GSH to trigger the release of drugs from nanoparticles, external stimuli have been proposed as well. The use of radiation such as microwaves and especially light appears among the more promising to introduce selectivity in the delivery of drugs and precisely control their release at different time points. In 2018, photoresponsive mesoporous silica nanoparticles (PMSN) were prepared as vehicles for the delivery of P-gp shRNA and photocaged prodrug of Dox [79] (Figure 25). To prepare this DDS, cationic poly[2-(*N*,*N*-dimethylaminoethyl)-methacrylate] was introduced onto the MSNs surface through a light-sensitive coumarin ester derivative linker to adsorb P-gp shRNA, whereas the photocleavable *o*-nitrobenzyl ester derivative-caged Dox was loaded into the inner pores of the PMSN. Once the NPs were internalized by MDR cancer cells, the release of the shRNA and Dox was demonstrated to be independently regulated by 405 and 365 nm light irradiations due to selectively cleavage of the coumarin and *o*-nitrobenzyl esters.

### 4.3. MSN Coated by Polycation with Targeting

The external layer of the polymeric coating of MSNs [50] can be decorated with a variety of ligands for targeted delivery [80]. In the context of dual delivery, four types of targeting have been implemented in the DDSs: Folic acid, hyaluronic acid (HA), lactobionic acid (LA), and peptides such as TAT.

#### 4.3.1. DDSs Decorated with Folic Acid

**Figure 26:**
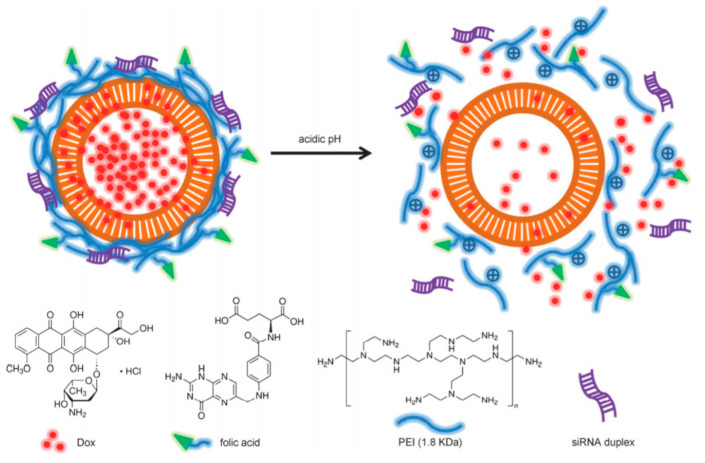
Illustration of targeted Dox/siRNA co-delivery by pH-responsive hollow silica nanospheres (HMSN). Reproduced from reference [81] https://doi.org/10.1002/chem.201302736. Copyright 2013 Wiley-VCH verlag GmbH & Co. KGaA, Weinheim.

In 2013, the group of Zhao reported the preparation of HMSN coated with PEI conjugated with FA for the dual delivery of Dox and siRNA against Bcl-2 protein, which shows anti-apoptotic activity [81] (Figure 26). The particles were first functionalized with phosphate groups by direct treatment with 3-(trihydroxysilyl)propylmethylphosphonate monosodium (TPMP). Subsequently, Dox was loaded inside the HMSN and the resulting solids were coated with FA conjugated PEI by means of electrostatic interactions. This functionalized polymer (PEI-FA) was obtained by reaction of FA with branched cationic PEI (1.8 kDa) through a Steglich coupling using EDC to form amide bonds between the carboxylic groups of folic acid and primary amine groups on polyethyleneimine. Finally, siRNA was absorbed onto the surface of the NPs. In vitro assessment of the DDS was conducted on HeLa and MCF-7 cell lines for comparison since HeLa cells present higher folic acid receptor expression than MCF-7. As anticipated, the endocytosis of the FA-decorated particles was found to be more successful in the HeLa cultures.

**Figure 27:**
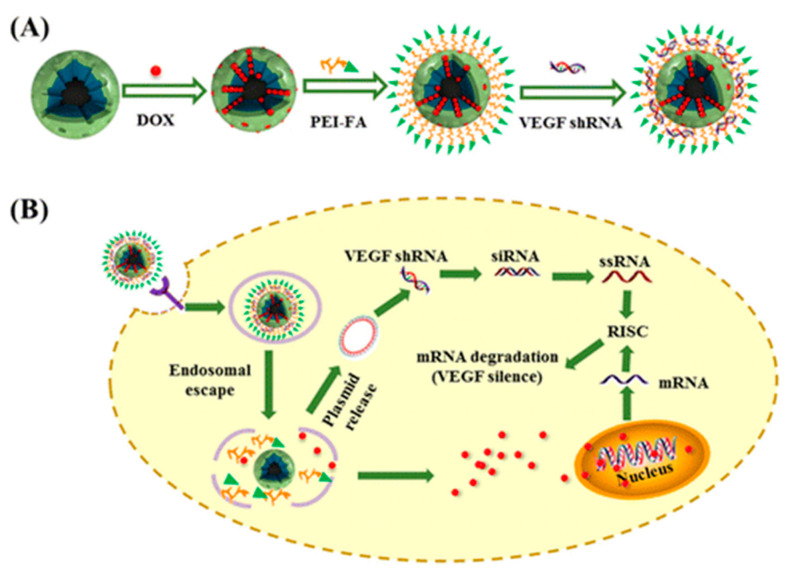
(**A**) Schematic of the M-MSN(DOX)/PEI-FA/vascular endothelial growth factor (VEGF) shRNA nanocomplexes; (**B**) schematic illustration showing the proposed delivery of DOX and VEGF shRNA-mediated by M-MSN/PEI-FA. Reproduced from reference [82] https://doi.org/10.1021/acsami.6b02963. Copyright 2016 American Chemical Society.

A similar DDS was introduced in 2016 by Liu and co-workers. In this study, magnetic-mesoporous silica nanoparticles were used for the selective co-delivery of Dox and vascular endothelial growth factor (VEGF) shRNA [82]. First, MSNs were coated with anionic groups by treatment with 3-trihydroxysilylpropyl methylphosphonate. Next, Dox was loaded into the pores and the voids were sealed by exposure of the particles to a PEI-FA copolymer. Finally, VEGF shRNA was incubated in the M-MSN(Dox)/PEI-FA suspension at various weight ratios for 30 min at room temperature to form the M-MSN(Dox)/PEI-FA/VEGF shRNA nanocomplexes by electrostatic absorption. In vitro antitumor activity assays revealed that HeLa cell growth was significantly inhibited. Quantitative PCR and ELISA assays revealed that M-MSN/PEI-FA/VEGF shRNA induced a significant decrease in VEGF expression as compared to cells treated with the control (Figure 27).

**Figure 28:**
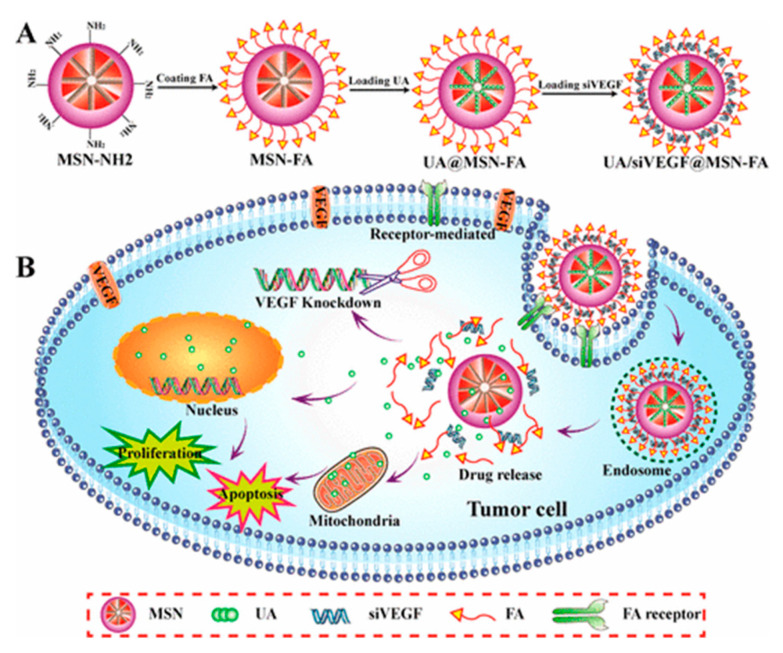
Schematic illustration of the (**A**) synthetic procedure for the preparation of UA/siVEGF@MSN-FA and (**B**) inhibiting effect on the proliferation of cancer cells. Reproduced from reference [83] https://pubs.acs.org/doi/10.1021/acs.jafc.7b03047. Copyright 2017. American Chemical Society.

One year later, Shao and co-workers reported a targeted drug delivery system to ferry ursolic acid (UA) and VEGF targeted siRNA (siVEGF) based on a MSN nanocarrier [83]. In concrete, aminated MSNs of 158 nm were used as vehicle. The preparation was straightforward. Firstly, FA was activated with *N*-hydroxysuccinimide (NHS) in the presence of EDC. The activated ester was then reacted with superficial amino groups of the MSNs. The resulting particles were loaded with UA in acetone as solvent. Finally, a suspension containing UA@MSN-FA and siVEGF was stirred to form complexes UA/siVEGF@MSN-FA. FR-overexpressing HeLa cells and FR-negative HepG2 cell lines were used to evaluate the in vitro cellular uptake and the cytotoxicity of MSN-FA nanoparticles. Furthermore, in vitro transfection showed that these particles could improve the transfection efficiency of siRNA and significantly inhibit the expression of VEGF proteins in HeLa cells (Figure 28).

**Figure 29:**
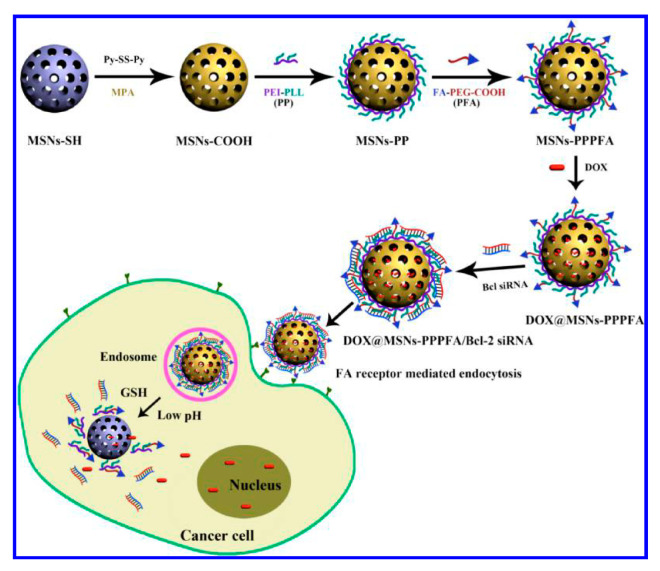
Co-delivery system of Dox and Bcl-2 siRNA based on MSNs modified by a PEI-PLL coating. Reproduced from reference [84] https://doi.org/10.1021/acs.jpcc.6b06759. Copyright 2016 American Chemical Society.

A redox sensitive DDS based on MSNs for the co-delivery system of Dox and Bcl-2 siRNA was presented by He in 2016 [84]. The polycation was tethered to the particle by means of covalent bonds sensitive to the presence of GSH. These smart nanoparticles were fabricated starting from thiol functionalized MSNs (Figure 29). NPs were first activated by exposure to 2,2′-dipyridyl disulfide (Py-SS-Py). Then, treatment with 3-mercaptopropionic acid yielded MSN-S-S-CO_2_H. The polycation utilized was a polyethylenimine−polylysine copolymer (PEI-PLL). After loading with Dox, the particles were reacted with targeting ligand folate-linked poly(ethylene glycol) (FA-PEG-COOH) using the NHS activation protocol to modify the polymer with FA through amide bonds. In vitro tests confirmed that the folate-conjugated MSNs-PPPFA showed significantly enhanced intracellular uptake in the folate receptor overexpressed MDA-MB-231 breast cancer cells than nontargeted counterparts and thus results in more Dox and siRNA being co-delivered into the cells.

#### 4.3.2. DDS Decorated with Hyaluronic Acid and Lactobionic Acid

**Figure 30:**
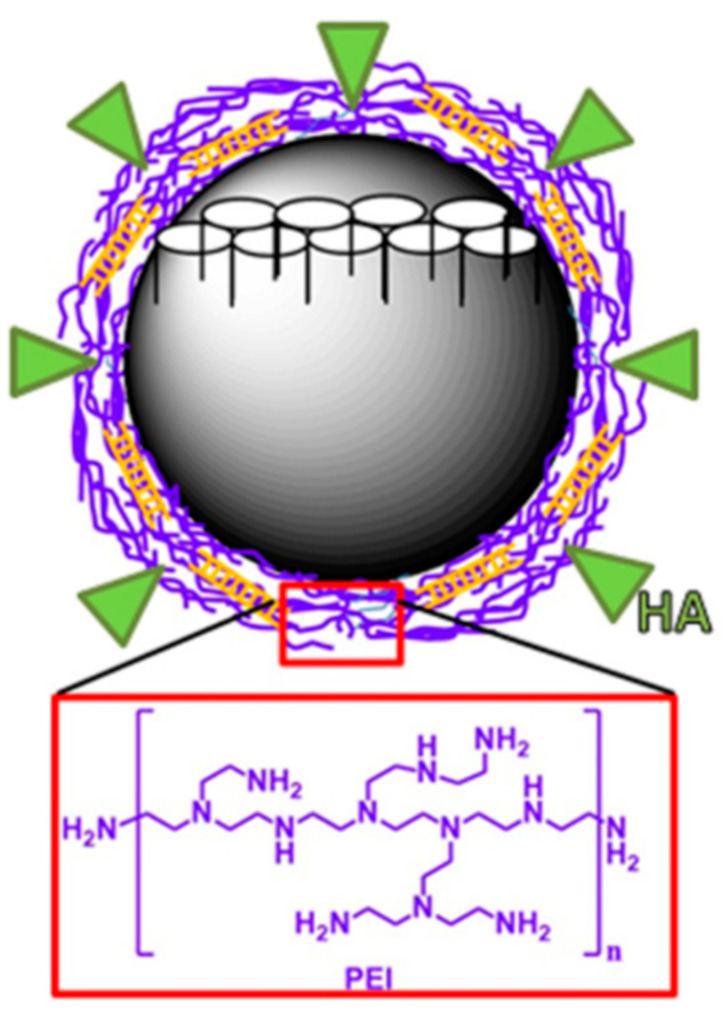
Hyaluronic acid (HA)-PEI coated MSNs for the delivery of siTWIST and cisplatin. Reproduced from reference [85] https://doi.org/10.1016/j.nano.2018.04.008. Copyright 2018 Elsevier.

The group of Glackin reported in 2018 the application of PEI coated MSNs for the delivery of siTWIST and cisplatin to epithelial ovarian cancer (EOC) cells [85] (Figure 30). To gain selectivity in the treatment, the authors conjugated HA to PEI using the EDC-NHS coupling protocol. The nanoparticles were coated with the polycation by electrostatic interaction with the phosphonate functionalized MSNs. HA conjugated mesoporous silica nanoparticles (MSN-HAs) carried siTWIST into target cells and led to sustained TWIST knockdown in vitro. Biological tests showed the inhibition of metastasis and multidrug resistance in Ovcar8-IP-eGFP cells. According to the authors, MSN-HAs resulting in significant selectivity, with better delivery efficiency and improved antitumor therapeutic effect as compared to current delivery systems. A similar approach was used the same year by Qin, Wen, and co-workers for the delivery of Dox and miRNA-31 [86].

**Figure 31:**
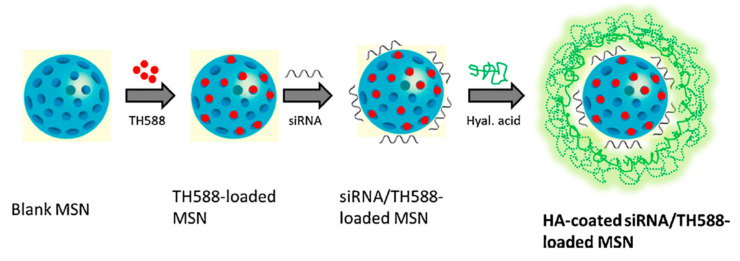
Schematic synthesis of a DDS for delivery of MTH1 inhibitor (TH287) and MDR1 siRNA via hyaluronic acid-based MSNs. Reproduced from reference [87] https://doi.org/10.1016/j.colsurfb.2018.09.076. Copyright 2019 Elsevier.

In 2019, Ding and co-workers prepared a DDS using HA coated MSNs with the aim to treat squamous cell carcinoma [87]. The DDS was loaded with TH287, a MTH1 inhibitor (6-(2,3-dichlorophenyl)-4-methylpyrimidine-2,4-diamine hydrochloride), and MDR1 siRNA (Figure 31). To synthetize the nano-vehicle, MSNs were functionalized with amino groups onto the surface of the particle by treatment with APTMS. Then, after loading the aminated particle with TH287, a HA solution was mixed with MSNs in a weight ratio of 2.5:10 (w/w) and incubated for 1 h. The negatively charged HA was binded to the positively charged MSNs via electrostatic interaction. Finally, the siRNA was loaded onto MSNs by mixing the siRNA and MSNs in 2-(*N*-morpholino)ethanesulfonic acid buffer (MES buffer) and allowed to incubate. Cytotoxicity and apoptosis assays showed that the combination of TH287+MDR1 siRNA was clearly more effective inducing the anticancer effect than MTH1 inhibitor alone. SiTMSN and HA-siTMSN significantly reduced the tumor burden compared to that of untreated control and free TH287.

**Figure 32:**
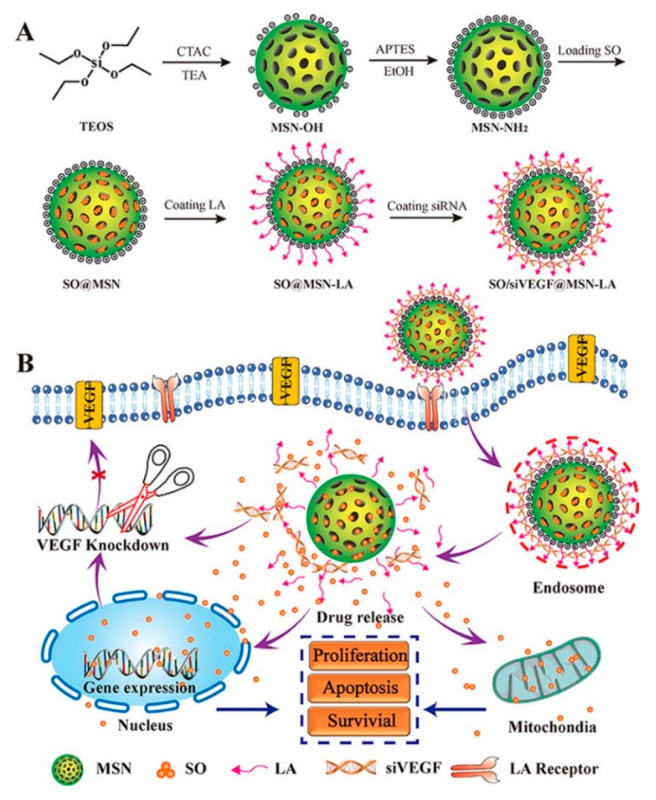
(**A**) Schematic illustration of synthesis procedure of sorafenib (SO)/VEGF targeted siRNA (siVEGF)@MSN-lactobionic acid (LA) NPs and (**B**) inhibiting effect on the proliferation of cancer cells. Reproduced from reference [88] https://doi.org/10.1016/j.ejps.2017.10.036. Copyright 2018 Elsevier.

A DDS intended for suppressing tumor progression in hepatocellular carcinoma for the co-delivery of sorafenib (SO) and siVEGF was communicated in 2018 by Shao and co-workers [88] (Figure 32). The nano-vehicle was fabricated starting from aminated MSNs. These particles were first loaded with sorafenib into the porous. Then, thanks to noncovalent interactions between the amino groups and lactobionic acid (4-O-β-D-galactopyranosyl-D-gluconic acid), the ligand was attached to the surface of the nanoparticle. LA was chosen as ligand to improve the selectivity of the device. This ligand can effectively bind asialoglycoprotein receptors (ASGPR) present in the membrane of liver cancer cells. Finally, siVEGF were loaded into the nano-carrier by electrostatic interaction. Cellular uptake, transfection and cell cytotoxicity of the DDS were investigated. In vitro testing demonstrated that SO/siVEGF@MSN-LA could not only induce S cell cycle arrest, enhance the cytotoxicity and improve the tumor target of SO and siVEGF, but also improve the siVEGF transfection efficiency in ASGPR-overexpressing Huh7 cells.

#### 4.3.3. DDS Decorated with Peptides

**Figure 33:**
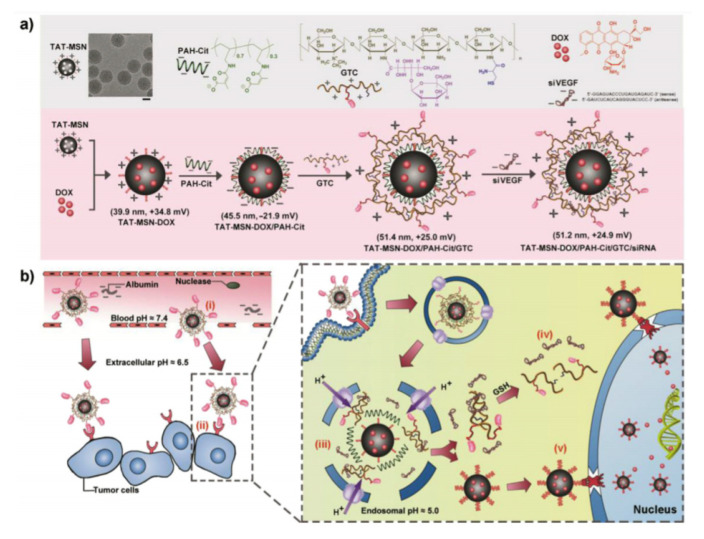
(**a**) Schematic illustration showing the formation of the MLNs via layer-by-layer self-assembly driven by the electrostatic coverage of PAH-Cit and GTC onto the TAT-MSN core (**b**) Schematic presentation of MLNs mediated delivery for DOX and siVEGF. Reproduced from reference [89] https://doi.org/10.1016/j.biomaterials.2015.05.001. Copyright 2015 Elsevier.

Cellular uptake and endosomal escape are basic steps that the DDS must go through to deliver the cargo. The fine tuning of these processes requires of a complex machinery. In 2015, Yin and co-workers proposed multi-layered nanocomplexes (MLNs) to optimize the smart co-delivery of drugs [89] (Figure 33). In this particular study, the nano-vehicle was intended for the co-delivery of Dox and vascular endothelial growth factor siRNA to QGY-7703 cells (hepatocarcinoma cells). The system reported consisted in an electrostatically self-assembled MLNs constructed by TAT peptide modified mesoporous silica nanoparticles (TAT-MSN) as the cationic core, poly(allylamine hydrochloride)-citraconic anhydride (PAH-Cit) as the anionic inner layer, and a galactose-modified trimethyl chitosan-cysteine (GTC) conjugate as the cationic outer layer to encapsulate siRNA. The stability of the layers at pH 7.4 and 6.5 protected siRNA from degradation in the blood and tumor microenvironment. The system showed selectivity toward tumor cells thanks to galactose ligands on the outer layers. Once the DDS was taken up, the endosomal/lysosomal acidity (pH 5.0) triggered the charge reversal of PAH-Cit, thereby inducing the disassembly of the MLNs and their escape to the cytosol. Cytoplasmic GSH further accelerated siRNA release through cleaving disulfide bonds in GTC layers.

**Figure 34:**
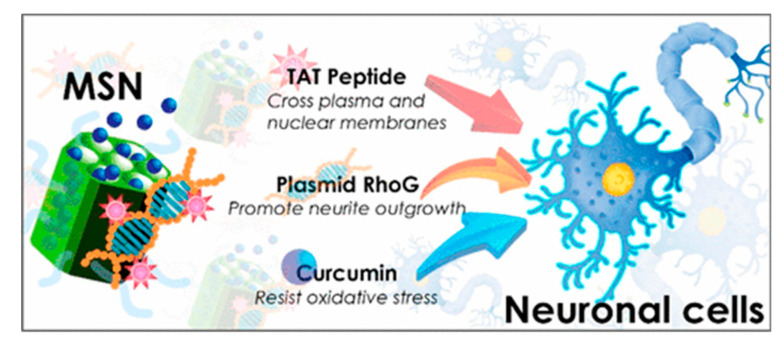
Co-delivery of curcumin and plasmid RhoG using MSNs. Reproduced from reference [90] https://pubs.acs.org/doi/abs/10.1021/acsami.9b02797. Copyright 2019 American Chemical Society.

Alternatively, TAT peptide can be as well attached to the DDS by electrostatic interaction. This scheme was followed by Wu, Chen and co-workers for the development of a carrier for the delivery of curcumin, a natural antioxidant, and plasmid RhoG-DsRed, which promotes neurite outgrowth [90] (Figure 34). The nanoparticles were prepared by co-condensation using APTMS derivatized with (fluorescein isothiocyanate (FITC)) and *N*-[(trimethoxysilyl)propyl]-*N,N,N*-trimethylammonium chloride (TMAC). Then, as-synthetized particles were loaded with curcumin. Separately, TAT peptide was adsorbed with negatively charged plasmid to provide a complex TAT/RhoG-DsRed. Next, TAT/RhoG-DsRed was incubated with Curcumin@MSN to render Cur@MSN-RhoG/TAT thanks to the ionic interaction of the positive surface of MSNs and the anionic plasmid/TAT. In this way, the plasmid RhoG-DsRed/TAT complex can act as a noncovalent gatekeeper.

**Figure 35:**
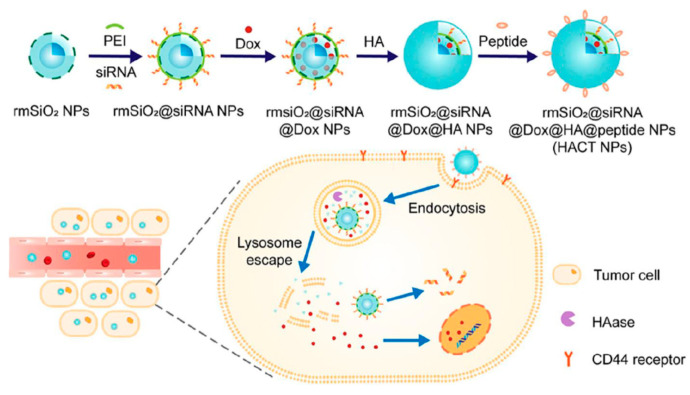
Schematic diagram of the “layer-by-layer assembly” strategy for the construction of HACT NPs with the cascade of two targeting agents (HA and peptide) and two cancer therapeutic agents (siRNA and Dox). Reproduced from reference [92] https://doi.org/10.3389/fchem.2020.00647. Copyright 2020 Frontiers in Chemistry.

An alternative to TAT is cell penetrating p-VEC peptide. In 2017, a complex DDS (HACT NPs) aiming at overcoming the drug resistance of CTGF-overexpressing breast cancer was prepared by co-embedding peptide (PEGA-pVEC) and HA as a targeting media for the delivery of siRNAs and Dox [91]. PEGA-pVEC peptide is composed of two components (Figure 35): A tumor-specific vascular homing peptide (PEGA), which allows identifying the higher expressive marker molecule, the membrane-bound proline-specific amino peptidase P (APasep) in the vasculature of breast cancer and a cell-penetrating peptide (p-VEC). Thus, the PEGA-pVEC peptide selectively leads NPs to accumulate in breast vasculature. Rattle mesoporous silica (rmSiO_2_) were used as inorganic carrier. PEI was absorbed onto the particles and mixed with siRNA. Then, Dox was bonded to the surface of the particles by electrostatic interaction. The complex formed this way was coated first with HA and, finally, with the targeting peptide PEGA-pVEC. Therefore, HA and PEGA-pVEC peptide played the triple role of targeting moieties, Dox gatekeeper, and protecting shell to prevent siRNA degradation by proteases. PCR and ELISA assays revealed that the system induced a significant increase in VEGF expression and the HUVECs were decreased on nanocomplexes-treated HeLa cells. Additionally, in vitro experiments were performed using MDA-MB-231 and MCF-7 as the control.

**Figure 36:**
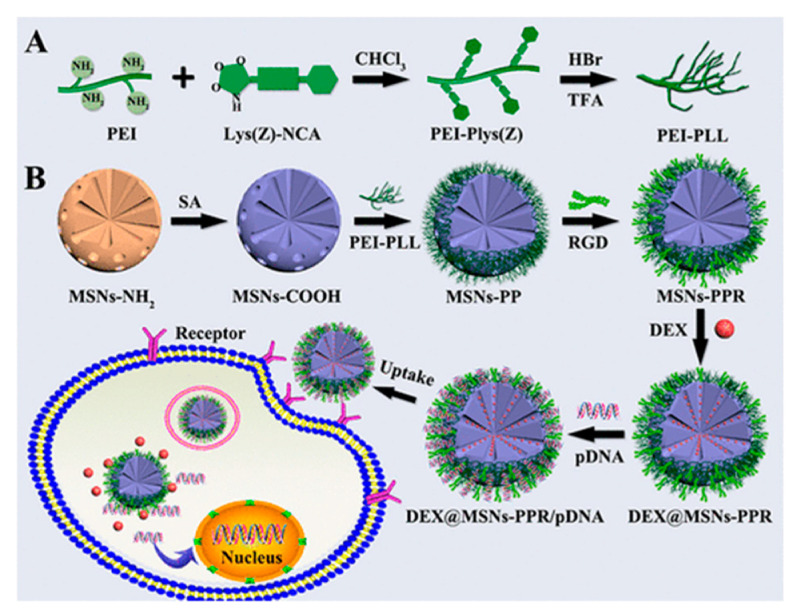
(**A**) Reaction scheme for the synthesis of PEI-PLL copolymers. (**B**) Schematic illustration for the preparation of PEI-PLL and RGD-conjugated MSNs-based nanocarrier and codelivery of pDNA and drug into cells. Reproduced from reference [93] https://doi.org/10.1021/acsbiomaterials.8b01110. Copyright 2019 American Chemical Society.

MSNs with RGD conjugated on their surface were designed for the simultaneous delivery of dexamethasone (DEX) and the BMP-2 gene [93] (Figure 36). This DDS was built using MSNs grafted with polylysine-modified polyethylenimine (PEI-PLL) copolymers with an arginine glycine-aspartate peptide anchored onto surface of MSNs. DNA plasmid of bone morphogenic protein-2 BMP-2 was attached to the surface of MSNs by adsorption. DEX, which is a drug used for the current treatment, was loaded inside the MSNs. The DDS was prepared starting from aminated MSNs derivatized with succinic acid to render MSN-CO_2_H. In parallel, polylysine-modified polyethylenimine (PEI-PLL) copolymers with various molecular weights PEI blocks were synthesized. The biological assessment of the particles demonstrated that the synthesized copolymer PEI-PLL-25k (synthesized using 25 kDa PEI) exhibited lower cytotoxicity and higher in vitro transfection efficiency than commercial PEI-25k (Mw = 25 kDa). Once in hand, these MSNs were grafted with PEI-PLL using NHS in the presence of EDC to render MSNs-PP. To introduce the RGD peptide the carboxyl group on RGD was activated with NHS and EDC and reacted with MSNs-PP to furnish MSNs-PPR. Next, DEX was loaded into MSNs-PPR. Finally, DEX-entrapped MSNs-PPR were loaded with pDNA.

### 4.4. SiRNA inside the Pores

**Figure 37:**
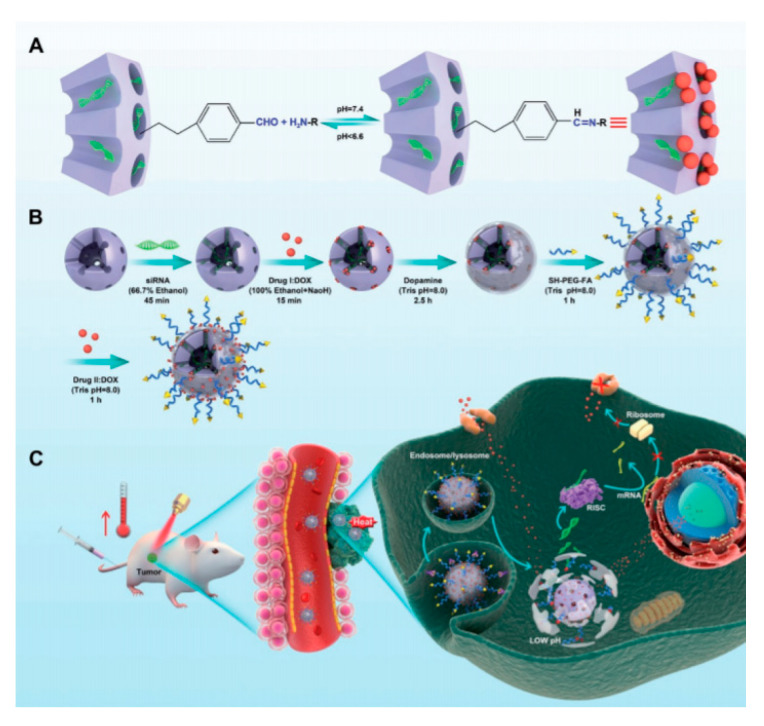
Schematic illustration of (**A**) the dynamic interaction between Dox and benzaldehyde via pH-sensitive benzoic–imine bond. (**B**) Synthetic route for the preparation of M-R@D-PDA-PEG-FA-D and (**C**) combined photothermal chemotherapy and gene targeted therapy of tumors. Reproduced from reference [94] https://doi.org/10.1002/adfm.201704135. Copyright 2017 Wiley-VCH verlag GmbH & Co. KGaA, Weinheim.

Considering that MSNs pores and siRNA have cylinder-like structure and the molecular size of siRNA (approximately 2 by 8 nm) is smaller than the average mesopore size of MSNs (2.3 nm by BJH method) it is theoretically feasible for siRNA molecules to enter into the mesopores of MSNs. In 2017, Zeng, Mei, and co-workers presented a DDS based on this possibility. In this work, P-gp siRNA was encapsulated inside the MSNs and the pores were capped with Dox linked to the NPs by imine bonds [94] (Figure 37). The outer layer was formed with polydopamine, which shows a photothermal activity, and the MSNs were decorated using FA to enhance the internalization of the system inside cells. Subsequently, in vitro, and in vivo antitumor experiments demonstrated the enhanced antitumor efficacy of the multifunctional nanoparticles.

## 5. Biological and Medical Applications

The practical application of the DDSs reported have been tested in vitro against a variety of cell lines. In the following Table 1, each system is described in terms of targeting ligand introduced in the DDS, the specific drugs and nucleic acid transported, and the cell line used to assess the therapeutic properties of the nanoparticles.

## 6. Conclusions

In this review, the design, synthesis, and application of MSNs for the delivery of multiple drugs in combination therapies have been presented.

MSNs share with other typical nano-platforms of drug delivery such as AuNP or liposomes low toxicity and biocompatibility. However, an advantage over these other supports in the design of DDS is their chemical versatility. The easy functionalization of the particles using common techniques of bioconjugation leads to the preparation of complex DDSs. This aspect is essential to translate any nano-vehicle into clinic since a reduction on the number of synthetic steps simplify the production process and the final scaling up.

Although the DDS presented in this review allow an efficient and selective delivery of multiple cargoes, still there are many issues to address. For instance, combination therapies rely on strict doses of the drugs to be effective. So, a precise spatio-temporal control of the dosage must be programmed. Another important issue is the intracellular delivery, since some drugs must be delivered to particular organelles within the cells for their optimal activity.

However, much have been accomplished in just a decade, this review has proven that. It is foreseeable a bright future for these sand nanorobots.

## Figures and Tables

**Table 1:** Description of the DDSs indicating the targeting moieties, loading, and cellular lines used to test the system.

DDS	Targeting	Cargo	Cellular Line	Ref.
Photoresponsive-MSN		ZnPc-Dox	HepG2	[35,36]
pH-triggered MSN		Dox/CPT	HeLa and U-87 MG	[38]
MSN		Dox/CPT/CdS	BxPc-3	[40]
Gossypol-capped MSN		Mitoxantrone/ gossypol	MCF-10A, MDA-MB-231	[42]
MSN		CPT/ 5-fluorouracil	MCF-7	[44]
g-CD-gated MSN		Rhodamine 6G/CPT calcein/Dox	HeLa cells, A549 cells	[46]
Triple stimuli-responsive MSN	FA	Dox/ 5-Fluoro-2-Deoxyuridine	DL, DLR, MCF-7, MCF-7R, K562, K562R	[47]
iRGD modified MSN	iRGD	Dox	GL261, CD3+ CD4+ CD8+ T	[48]
Photoresponsive- MSN		Cisplatin Prodrug/Chlorin e6	A549	[49]
pH/redox-triggered MSN		doxorubicin/paclitaxel	BT549, MCF-10A	[53]
Pyridylthiol-terminated MSN	LHRH peptide	Doxorubicin/cisplatin/MRP1 and BCL2 mRNA	A549	[56]
Redox-responsive MSN		Dox/ Bcl-2 siRNA	MCF-7, HEK 293	[57]
MSN-PEI		Doxorubicin, P-gp siRNA	KB-V1	[58]
MSN-PEI		Dox/ P-gp, MRP1, ABCG2, Bcl-2, cMyc, PXR siRNA	MCF-7	[59]
MSN-PEI		Dox/ MDR1 siRNA	KBV cells	[60]
MSN MSN-PEI-PEG		Dox/ (P-g)-si-RNA	MDA-MB-231 A549	[62]
		Dox	KB-31	[63]
MSN-PEG	FA, TTA	Dox	B16-F10, HeLa, MCF-7	[65]
pH-sensitive MSN		Indomethacin/ docetaxel (DTX)	B16F10, HepG	[66]
Mesoporous core-shell silica nanoparticles		TWITS siRNA/ daunorubicin	Ovcar8	[67]
ICP-MSN		Dox/ Bcl-2 siRNA	A2780/AD human ovarian cancer cells	[68]
Polycation-modified MSN		Chloroquine	B16F10 murine melanoma cell	[69]
FMSN		pNurr1/ siRex1	iPSCs	[70]
MSN		Dox	PANC-1	[30]
FMSN		Cisplatin/siTWIST	Ovcar8-IP	[70]
Modified large pore MSN Dendritic MSN		Chloroquine/siRNA	KHOS	[71,72]
		Dox/ Survivin shRNA	QGY-7703	
				[75]
MSN based on ZIF-8		Dox/siRNA	MCF-7 SKOV-3 ADR	[74]
Redox-sensitive HMSN		Dox/ P-gp modulator siRNA	MCF-7 ADR	[79]
Redox-responsive and self-destructive MSN		Plasmid p53	HepG2 C6	[78]
Photoresponsive-MSN		Dox/shRNA(P-gp)	MDR HepG2 ADR	[80]
Light sensitive coumarin HMSN/PEI-FA	FA	Dox/siRNA(Bcl-2)	HeLa MCF-7	[81]
M-MSN/PEI-FA/VEGF shRNA		Dox/shRNA(VEGF)	HeLa	[82]
UA/siVEGF@MSN-FA	FA	Ursolic acid/siRNA(VEGF)	HepG2 HeLa	[83]
Redox sensitive MSN -PPPFA	FA	Dox/Bcl-2-siRNA	MDA-MB-231 breast cancer cells	[84]
MSN-HA	HA	cisplatin/ siTWIST	Ovcar8-IP-eGFP	[85]
HA-siTMSN	HA	TH287/MDR1 siRNA	CAL27	[87]
SO/ siVEGF@MSN-LA	HA Lactobionic acid	sorafenib/SiRNA(VEGF)	ASGPR-overexpressing Huh7	[88]
MLNs	TAT	Dox/siRNA(VEGF)	QGY-7703	[89]
Cur@MSN-RhoG/TAT	TAT	curcumin/ RhoG-DsRed	Neuro-2a	[90]
rmSiO_2_	PEGA-Pvec HA	Dox/siRNA(CTGF)	MDA-MB-231 MCF-7 HeLa	[91]
MSN-RGD conjugated	RGD	DEX/pDNA protein-2 BMP-2	BMSC	[93]
Photoresponsive-MSN	FA	polydopamine/Dox/P-gp siRNA	MCF-7 ADR	[94]
